# Sizeable net export of base cations from a Carpathian flysch catchment indicates their geogenic origin while the ^26^Mg/^24^Mg, ^44^Ca/^40^Ca and ^87^Sr/^86^Sr isotope ratios in runoff are indistinguishable from atmospheric input

**DOI:** 10.1007/s11356-024-32866-1

**Published:** 2024-03-18

**Authors:** Martin Novak, Yulia V. Erban Kochergina, Alexandre V. Andronikov, Chris Holmden, Frantisek Veselovsky, Vaclav Kachlik, Jakub Hruška, Frantisek Laufek, Tomas Paces, Arnost Komarek, Ondrej Sebek, Marketa Stepanova, Jan Curik, Eva Prechova, Daniela Fottova, Irina E. Andronikova

**Affiliations:** 1 Department of Environmental Geochemistry and Biogeochemistry, Czech Geological Survey, Geologicka 6, 152 00 Prague 5, Czech Republic; 2 Department of Rock Geochemistry, Czech Geological Survey, Geologicka 6, 152 00 Prague 5, Czech Republic; 3 Saskatchewan Isotope Laboratory, Department of Geological Sciences, University of Saskatchewan, 114 Science Place, Saskatoon, SK S7N 5E2 Canada; 4 Geology Department, Faculty of Science, Charles University, Albertov 6, 118 21 Prague 2, Czech Republic; 5 Faculty of Mathematics and Physics, Charles University, Sokolovska 49, 186 75 Prague 8, Czech Republic

**Keywords:** Magnesium, Calcium, Strontium, Isotopes, Nutrient imbalances, Forest catchment

## Abstract

Nutrient imbalances may negatively affect the health status of forests exposed to multiple stress factors, including drought and bark beetle calamities. We studied the origin of base cations in runoff from a small Carpathian catchment underlain by base-poor flysch turbidites using magnesium (Mg), calcium (Ca) and strontium (Sr) isotope composition of 10 ecosystem compartments. Our objective was to constrain conclusions drawn from long-term hydrochemical monitoring of inputs and outputs. Annual export of Mg, Ca and Sr exceeds 5-to-15 times their atmospheric input. Mass budgets per se thus indicate sizeable net leaching of Mg, Ca and Sr from bedrock sandstones and claystones. Surprisingly, δ^26^Mg, δ^44^Ca and ^87^Sr/^86^Sr isotope ratios of runoff were practically identical to those of atmospheric deposition and soil water but significantly different from bedrock isotope ratios. We did not find any carbonates in the studied area as a hypothetical, easily dissolvable source of base cations whose isotope composition might corroborate the predominance of geogenic base cations in the runoff. Marine carbonates typically have lower δ^26^ Mg and ^87^Sr/^86^Sr ratios, and silicate sediments often have higher δ^26^Mg and ^87^Sr/^86^Sr ratios than runoff at the study site. Mixing of these two sources, if confirmed, could reconcile the flux and isotope data.

**Supplementary Information:**

The online version contains supplementary material available at 10.1007/s11356-024-32866-1.

## Introduction

Magnesium (Mg) and calcium (Ca) are essential nutrients performing a variety of functions in the biosphere (Marschner 1995). Magnesium serves as a coordinating cation in chlorophyll and activates enzymes needed for the synthesis of organic molecules, while calcium functions as an intracellular messenger and stabilizes cell walls (Sterner 2002). During pedogenesis, the sources of nutrients change. As soils develop on freshly exposed bedrock, geogenic elements are gradually lost (Wiegand et al 2005; van der Heijden et al 2014; Court et al 2021; Bukoski et al 2021; von Blanckenburg et al 2021; Basdedios et al 2023). When the rates of hydrological export of geogenic nutrients, such as Ca, Mg, K, and P, are higher than the rates of dissolution of bedrock minerals, atmospheric inputs gradually replace rock-derived nutrient sources and help to sustain the productivity of forests. This process has been documented for highly weathered soils in humid, warm regions (Chadwick et al 1999), but also inland ecosystems in the temperate climatic zone have been shown to store substantial amounts of atmospheric nutrients. For example, Miller et al (1993) estimated that 50 to 60% of base cations in the organic soil exchangeable pool and biomass in the Adirondack Mts. (NY, USA) have an atmospheric origin. Belanger and Holmden (2010) calculated that 45 to 90% of Ca present in a Canadian catchment originated from atmospheric deposition. Maher and von Blackenburg (2023) showed that increased water export or decreased regolith thickness due to erosion diminishes the total inventory of nutrients, leading to lower rates of recycling via the plant-available soil reservoir and lower plant growth. Carbonate minerals are associated with considerably faster dissolution kinetics than silicates (Chen et al 2023, and references therein). In the long term, phosphorus (P) is believed to be the master regulator of ecosystems, but Ca and Mg are more mobile than P and may be depleted more rapidly (Chadwick et al 1999). Organic charges that dominate the cation exchange capacity in soils are characterized by a higher affinity for Ca than for Mg (Oursin et al 2023), and, therefore, leaching of Mg through the soil profile is faster, compared to Ca (Maguire and Cowan 2002).

Here we present a combined Ca–Mg isotope and mass-balance study of a small forested catchment near the border between the Czech Republic and Poland. Our main objective was to constrain the provenance of Ca and Mg in surface runoff from a steep-slope headwater site on flysch bedrock situated in a highly industrialized part of Silesia. Over the past 60 years, this Central European region has experienced numerous types of stress, including acid rain (*ca.* 1970–1995; Hruška et al 2023), elevated trace metal deposition (since the 1950s; Voldrichova et al 2014), bark-beetle infestations (1992–1996; 2015–2023; Soukhovolsky et al 2022) and climate warming (droughts between 2014 and 2019; Oulehle et al 2021). In the Czech Republic alone, spruce die-back due to anthropogenic acidification affected an area of 500 km^2^, while conifer stands disintegration due to the present-day bark beetle calamity has occurred on an area of 800 km^2^ (Zahradnik and Zahradnikova 2023). Atmospheric deposition of alkaline earth elements increased in Central Europe after 1950 as a result of elevated dust emissions from numerous Soviet-style thermal power plants. The resulting replenishment of base cations in ecosystems via wet and dry deposition diminished in the mid-1990s due to technological upgrades in the cluster of soft-coal burning power plants located upwind from the studied region (Kopacek et al. 2016).

Abundance ratios of stable isotopes of Mg and Ca can be used not only as tracers of their inputs into catchment reservoirs but also provide insights into mixing history between solutes, secondary mineral formation, and the recycling of atmospheric and lithogenic inputs by vegetation (Bullen et al 2004; de Villiers et al 2005; Tipper et al 2006, 2008, 2012; Pogge von Strandmann et al 2008; Jacobson and Holmden 2008; Wimpenny et al 2011; Ma et al 2015; Oehlerich et al 2015; Schmitt 2016; Fahad et al 2016). Isotope ratios are reported in δ values as per mil (‰) deviations in the composition of the sample relative to a standard. The commonly used δ^26^Mg values refer to ^26^Mg/^24^Mg ratios, whereas δ^44^Ca values refer to ^44^Ca/^40^Ca ratios. Isotopically heavy samples are characterized by high δ values, while isotopically light samples have low δ values.

Stable isotope systematics of Mg and Ca in the critical zone (defined as the space between the outer extent of vegetation and the lower limits of groundwater; Brantley et al. 2005) exhibit some similarities. High-temperature processes involving both elements are associated with smaller isotope fractionations than those occurring under lower temperatures (Antonelli et al 2023). Bedrock weathering per se does not fractionate Mg and Ca isotopes (Ryu et al 2011; Cobert et al 2011). Present-day sea water is characterized by relatively light Mg compared to bulk crust and mantle (Guo et al 2019). One exception is represented by marine carbonates preferentially accumulating Mg that is isotopically lighter than the sea water. In contrast, a great majority of geological and environmental samples contain isotopically lighter Ca compared to sea water. On the continents, two well-constrained Mg and Ca isotope fractionation processes occur (Wiggenhauser et al 2022; Cai et al 2022). These are associated with the formation of secondary minerals and uptake by plants (Schmitt 2016; Tipper et al 2016; Teng 2017). River waters typically carry lower δ^26^ Mg values than the upper crust because clays preferentially bind isotopically heavy Mg (Brewer et al 2018; Ryu et al 2021; Li et al 2021; Fan et al 2023). Magnesium isotope composition of bulk clay, however, is site-specific, depending on the proportion of isotopically heavy structural Mg and isotopically light exchangeable Mg adsorbed on the phyllosilicate surfaces (Opfergelt et al 2012, 2014; Fries et al 2019; Hindshaw et al 2020; Zhao et al 2022a). The precipitation of secondary carbonates is an important mechanism driving δ^26^Mg of the dissolved load in rivers to higher values (Zhao et al 2019). Some secondary minerals preferentially bind isotopically light Ca, leaving behind soil solutions and runoff water with isotopically heavy Ca (Brazier et al 2019). Plants often prefer isotopically heavy Mg relative to the nutritive substrate (Black et al 2008; Chapela Lara et al 2017; Schuessler et al 2018). From roots and stem wood toward branches and foliage Mg becomes isotopically lighter (Bolou-Bi et al 2012). In contrast, during assimilation, isotopically light Ca preferentially accumulates in the plant tissues, with Ca in the residual growth medium becoming isotopically heavy. From roots upward to leaves or needles, tree organs typically contain isotopically heavier Ca (Schmitt 2016, and references therein).

In the current study, Ca isotope data have been complemented by ^87^Sr/^86^Sr isotope determinations. These two alkaline earth elements have similar ionic radii and charge and, for many years, were assumed to behave similarly in the critical zone (Åberg et al. 1990; Bedel et al 2016; Burger and Lichtscheidl 2019). Strontium isotope ratios could then serve as a proxy of Ca cycling. Indeed, many studies showed that Sr isotope ratios are a useful tracer of hydrological processes (Nuruzzama et al 2020; Demonterova et al 2022). Recent work, however, has indicated that Ca and Sr cycling in biological processes is de-coupled. Strontium isotope data in Ca-nutrient studies should thus be used with caution (Drouet et al 2005; Brenot et al 2008a,b; Ryan et al 2018; Bouchez and von Blackenburg 2021; Nguyen et al 2023).

Cenki-Tok et al (2009) pointed out that “determination of the atmospheric contribution of base cations to catchment surface water using mass budget calculations that are based only on annual element fluxes must be taken with caution.” In the current study, we focused on a comparison between Mg, Ca and Sr input–output budgets, which represent the traditional “black-box” approach, and stable isotope fingerprinting which additionally reflects within-catchment processes. We hypothesized that the Mg, Ca and Sr isotope signatures of runoff would mainly reflect the isotope composition of bedrock in the case of a large net hydrological export of the studied elements. In the case of net accumulation of atmospheric base cations in the catchment and/or a balanced mass budget, the isotope composition of runoff would indicate a mixture of geogenic and atmospheric sources. We also hypothesized that shallow soil water would mostly carry isotope signatures of present-day atmospheric Mg, Ca and Sr. The isotope signatures of soil water would differ from the isotope signatures of bulk soil and runoff, both of which would, under net Mg, Ca and Sr export, converge to those of bedrock. Collectively, the flux and isotope data on base cations in small catchments can help to evaluate the sustainability of forests in the present era of global change.

## Methods

### Study site

**Fig. 1:**
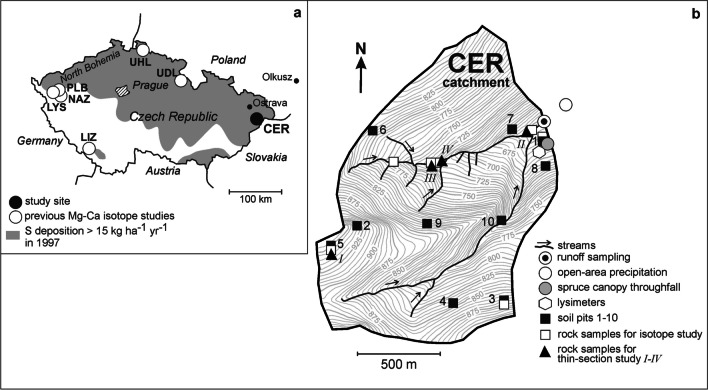
Study site location (*a*), and sampling scheme in the Cervik (CER) catchment (*b*). Previous Mg, Ca and Sr isotope studies in headwater catchments in panel (*a*) are marked by open circles (Novak et al 2020a, 2020b, 2020c, 2021, 2023a, 2023b). Sulfur deposition data by Czech Hydrometeorological Institute

The small catchment Cervik (CER) is located in the Moravian-Silesian Beskydy Mts. in the eastern Czech Republic (Central Europe), close to the borders with Poland and Slovakia (Fig. 1; Table 1; Adamova 1986; Novak et al. 1996, 2005; Oulehle et al 2017; 2021; Vicha 2019). Geologically, the area belongs to the folded Upper Cretaceous flysch complexes of the Silesian Unit (Outer Western Carpathians; Golonka et al. 2019). The bedrock of the studied catchment is formed by sandstone with subordinated amounts of claystone. It is part of the Istebna Formation (Campan–Paleocene; Appendix I; for geological sketch, *see* Fig. S1 of the Electronic Annex). North and east of CER, the older Godula Formation crops out. The maximum elevation of the catchment is 961 m. Deluvial sediments, along with cambisol and stagnosol soils, cover nearly 70% of the surface of the catchment (Fig. S1). The soil depth fluctuates between 40 and 80 cm. Mature Norway spruce (*Picea abies*) stands cover 88% of the catchment’s area, with clearings and young deciduous trees encompassing the remaining 12%. Atmospheric deposition of sulfur (S), directly measured in the catchment since 1994, reached 35 kg ha^−1^ yr^−1^ in 1996 and decreased to 6 kg ha^−1^ yr^−1^ in 2021 (Oulehle et al 2021; Fig. S2). The nearly sixfold decrease in annual S deposition over the 28-year time period followed a peak in nation-wide S industrial emission and atmospheric deposition rates in the late 1980s (Hruška et al 2002; Oulehle et al 2006; Hunova et al 2014, 2018; Hunova 2020). Acidifying compounds deposited at the study site originated from (i) North Bohemian soft-coal burning power plants located 300 km to the west (Novak et al 2000), (ii) the Silesian industrial area near Ostrava (eastern Czech Republic, stone coal and metallurgy; 40 km to the north) and (iii) Olkusz industrial area (southern Poland, base metals and thermal power plants; 140 km to the northeast; Fig. 1a; Buzek et al 2017, 2023; Prechova et al 2020, 2023). After 1994, reactive nitrogen (N_r_) deposition at CER fluctuated between 5 and 14 kg ha^−1^ yr^−1^ (Oulehle et al 2021; Fig. S2) and lacked clear-cut temporal trends. The mean pH of spruce throughfall was slightly lower than the mean pH of open-area precipitation in most years. During the 1994–2021 time period, pH of both spruce throughfall and open-area precipitation increased almost linearly from 3.9–4.4 to 5.3–5.5. The pH of runoff (mean of 6.8 for the years 1994–2021) was significantly higher than pH of atmospheric deposition. In contrast to atmospheric deposition, pH of runoff did not increase over time.

**Table 1:** Study site characteristics

Site	Cervik (CER) Czech Republic
Location/Coordinates	Beskydy Mts., Carpathians / 49°27´N, 18°23´E
Catchment area (ha)	181
Bedrock	sandstone, claystone, conglomerate
Geological unit	flysch-Istebnan sandstone
Soil type	cambisols, stagnosols
Elevation (m)	637–961
Mean annual temperature (ºC)	+ 6.8
Mean annual precipitation (mm)	1155
Mean snow cover (days)	112
Evapotranspiration (mm)	519
Mean pH of rainfall^*^	5.5
Mean Cl^−^ concentration in rainfall^*^ (mg L^−1^)	0.48
Mean pH of runoff^*^	6.8
Mean CEC^*^ (meq kg^−1^)	88
Mean BS^*^ (%)	16
Mean ANC^*^ (µeq L^−1^)	240
Vegetation	Norway spruce (*Picea abies*) 88%, clearings 12%
Tree age (yr)	50–70

^*^ Geomon Database, Oulehle et al. 2021

### Sampling

The locations of CER sampling sites are in Fig. 1b. The Mg and Ca input–output flux estimates are based on monthly sampling of open-area precipitation, spruce canopy throughfall, and surface runoff in hydrological years 1994–2021. Strontium input–output fluxes are based on monthly sampling in hydrological years 2014–2021. Samples of both types of atmospheric deposition and runoff for Mg, Ca, and Sr isotope analysis were collected between November 2020 and October 2021 (Tab. S1 and S2 in the *Electronic Annex*). Precipitation collectors were described by Fottova and Skorepova (1998). Soil water for δ^26^Mg, δ^44^Ca and ^87^Sr/^86^Sr determinations was sampled monthly between June and November 2021. The mineral soil solutions were collected in triplicate by *Prenart* suction lysimeters from a depth of 60 cm and pooled bi-monthly prior to analysis (Tab. S1 and S2).

Solid samples included bulk rock, forest floor (L, HF), soil from the depth intervals 0–20, and 20–40 cm, spruce xylem, class-1 spruce needles, and spruce roots < 2-mm in-diameter. Each sample type, except for bulk bedrock, was collected at 10 to 20 sites around soil pits no. 1, 3 and 5 (solid squares in Fig. 1b and Fig. S1) and combined to form three replicates. Bedrock samples (open squares in Fig. 1b and Fig. S1) were collected as follows: Thirty sandstone samples were collected in the area of soil pits no. 1, 3 and 5 and combined to form three replicates for isotope analysis. Ten claystone samples were collected from each of the three small outcrops in the stream and combined to form three replicates for isotope analysis. Conglomerate was identified and sampled only in one outcrop. Bedrock for a thin-section study was sampled at sites *I*, *II*, *III* and *IV* (solid triangles in Fig. 1b and Fig. S1). Spruce xylem was sampled 1.4 m above ground using a tree-ring corer. Tables S1 and S2 give the numbers of individual sample types analyzed for Mg, Ca, and Sr isotopes.

Pool-size inventory of Mg and Ca in soil was based on 10 soil pits excavated below a 0.5-m^2^ area in 2014 (Fig. 1b). Pool sizes of Mg and Ca were calculated per unit area for aboveground and belowground vegetation, forest floor, and four soil depth intervals (0–10, 10–20, 20–40, and 40–80 cm).

### Sample processing and analysis

Detailed methodology of sample processing and analysis can be found in Novak et al (2023a). Here, we briefly summarize individual steps.

#### Homogenization

Bedrock fragments (three composite samples of sandstone, three composite samples of claystone, and one sample of conglomerate) were ground in a jaw crusher and milled. The samples were used for Mg, Ca and Sr isotope analysis and whole-rock silicate analysis. The < 2-mm soil fraction was prepared for isotope analysis by sieving and milling to a < 60-μm grain size. Forest floor samples were homogenized without sieving. Stratified soil samples from 10 soil pits (Fig. 1b) were air-dried, sieved (< 2 mm) and homogenized. Exchangeable soil pools of Mg and Ca were determined using BaCl_2_ extracts (Oulehle et al 2010). In contrast, total Mg and Ca pools in aboveground vegetation (spruce xylem and needles) and belowground vegetation (spruce roots) were used for the inventory.

#### Solid sample dissolution

Samples of bulk bedrock and soil were dissolved in HF and HClO_4_ and re-dissolved in concentrated HNO_3_. Biomass/organic-rich materials were ashed at 550 °C for 8 h, digested in H_2_O_2_ and HNO_3_, and further treated using the same steps as in the case of bedrock and soil.

#### Sample purification and mass spectrometry

For the chromatographic separation of Mg, we used the methodology by Pogge von Strandmann et al (2011) modified to a one-column procedure (Pokharel et al 2017). Separation of Ca was carried out using the methodology described by Holmden (2005) and Holmden and Belanger (2010). Strontium processing was performed according to Erban Kochergina et al (2022).

The Mg isotope ratio measurements were conducted using a *Neptune* MC-ICP-MS (*Thermo Scientific*). Details are in Pokharel et al (2017). The Ca and Sr isotope ratio measurements were conducted using a *Triton* Thermal Ionization Mass Spectrometer (TIMS; *Thermo Scientific*) using the methods described by Holmden and Belanger (2010) and Erban Kochergina et al (2022), respectively.

The reproducibility of the δ^26^Mg measurements was ± 0.07 ‰ (2 SD) based on repeated analyses of NIST SRMs 1640a, 1515, and 2709a, USGS standard BHVO-2, and IAPSO standard seawater. The reproducibility of the δ^44^Ca measurements was ± 0.07 ‰ (2 SD) based on repeated analyses of NIST SRMs 915a and 915b (Tab. S3). Repeated analysis of the NBS 987 Sr isotope standard gave an ^87^Sr/^86^Sr ratio of 0.710243 ± 0.000007 (2 SD). The reproducibilities were similar to those reported by Pin et al (2014), Teng et al (2017), Shalev et al (2018) and Janousek et al. (2022; Tab. S3).

#### PXRD whole-rock analysis

Powder X-ray diffraction data were collected on a *Bruker D8* Advance powder diffractometer in a Bragg–Brentano geometry. A qualitative phase analysis was performed using the DIFFRAC.Eva software (Bruker 2015). The quantitative phase analysis was carried out using the Rietveld method (Topas 5 Program; Bruker 2014). The detection limit of the method was between 0.2 and 1.0 wt.

#### Analysis of liquid samples

Element concentrations in natural solutions were determined by FAAS (Mg and Ca), Q-ICP-MS (Sr), and ICP OES (Ba and Rb). Aliquots for Mg and Ca isotope analysis were filtered (0.45 μm), evaporated to dryness and treated with concentrated HNO_3_. Rare solutions where solid residues occurred were re-dissolved in concentrated H_2_O_2_ and HNO_3_. Water samples for Sr isotope analysis were filtered, acidified with HNO_3,_ evaporated to dryness, and the residue, if present, was dissolved in a H_2_O_2_-HNO_3_ mixture, evaporated to dryness and dissolved in HCl.

#### Carbon analysis

Carbonate C was released from the sample by reaction with concentrated phosphoric acid. Dried CO_2_ was analyzed in an ELTRA CS 500 Analyzer with infrared detection (precision of 6%). Total C and S were analyzed following the thermal decomposition of the sample (precision of 5 and 4%, respectively).

#### Mg and Ca inventories and input–output budgets

Annual catchment-level budgets of the studied elements were calculated as atmospheric inputs minus runoff outputs. Atmospheric input fluxes were weighed by the percentages of the catchment’s surface covered by spruce stands and clearings, respectively.

#### Statistics, GIS and mixing models

Comparisons of Mg, Ca, and Sr isotope compositions of individual sample types were based on a one-way analysis of variance and Tukey’s multiple comparisons method (Tukey 1953). The estimated slopes and *p*-values of decreasing fluxes of dissolved Mg and Ca were based on a linear model with autoregressive errors over time.

ArcGIS, version 10.3, was used to average slopes in the catchment. The slope raster was generated from a digital elevation model (DEM; spatial resolution of 1 m). A model by Capo et al. (1998) was used to estimate the contributions of possible major mixing endmembers to base cations in runoff (Appendix II).

## Results

### Mg, Ca and Sr isotope systematics

**Fig. 2:**
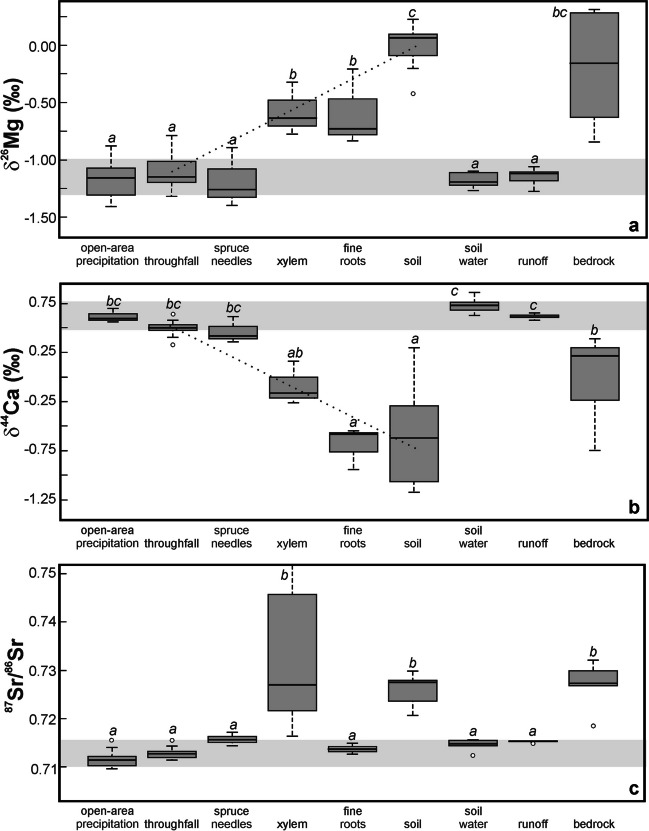
Boxplots depicting Mg, Ca and Sr isotope systematics in the CER catchment. Boxes include the second and third quartile, median is marked by a horizontal line within each box. For each of the studied elements, a horizontal gray band illustrates statistically indistinguishable isotope ratios of atmospheric deposition, shallow soil water, and runoff. Dotted lines in panels (*a*) and (*b*) may suggest mixing of Mg and Ca, between spruce canopy throughfall and soil to form spruce xylem as well as assimilation-related isotope fractionations. Different letters in superscript denote sample types that are significantly different (*p* < 0.05)

All three studied elements exhibited an analogous isotopic relationship among four types of water samples. For each of the Mg, Ca, and Sr systems, the isotope ratios of open area precipitation, spruce throughfall, soil water and runoff were statistically indistinguishable (*p* > 0.05; Fig. 2). In contrast, the isotope composition of Mg, Ca and Sr in bedrock was significantly different from the isotope composition of Mg, Ca, and Sr in runoff and soil water (*p* < 0.05). In Fig. 2a, b, c, the horizontal gray bands highlight the contrast between the isotope composition of the studied base cations in bedrock and in water samples.

The δ^26^Mg, δ^44^Ca and ^87^Sr/^86^Sr ratios of spruce needles were statistically indistinguishable from δ^26^Mg, δ^44^Ca and ^87^Sr/^86^Sr ratios of precipitation and throughfall (*p* > 0.05). The δ^26^Mg values of spruce roots and xylem were plotted between those of atmospheric deposition and bulk soil (Fig. 2a). Bulk soil δ^26^Mg values were indistinguishable from bedrock (*p* > 0.05). Similarly, the δ^44^Ca values of spruce roots and xylem plotted between the δ^44^Ca values of atmospheric deposition and bulk soil (Fig. 2b). The δ^44^Ca values of soil were the lowest in the whole studied system, significantly lower (*p* < 0.05) than those of bedrock. In the case of the ^87^Sr/^86^Sr ratios, fine roots were indistinguishable from soil water. At the same time, there was no significant difference between the ^87^Sr/^86^Sr ratios of xylem, bulk soil and bedrock.

The time-series in Fig. S3a,b,c show nearly constant δ^26^Mg, δ^44^Ca and ^87^Sr/^86^Sr ratios of runoff (their temporal variability was close to the uncertainly of mass spectrometric measurements). The variability in δ^26^Mg and δ^44^Ca values of atmospheric deposition was also relatively low (within 0.6 ‰ and 0.4 ‰, respectively). The variability in ^87^Sr/^86^Sr isotope composition of atmospheric deposition was large (0.08). No seasonality in δ^26^Mg, δ^44^Ca and ^87^Sr/^86^Sr ratios of liquid samples was observed. An ^87^Sr/^86^Sr *vs.* 1/Sr concentration plot showed a relatively large negative correlation (Fig. S3d; *R*^*2*^ = 0.72). The two extreme values in Fig. S3d *(bottom right)* corresponded to the wettest and driest month of the year respectively (April and September).

**Fig. 3:**
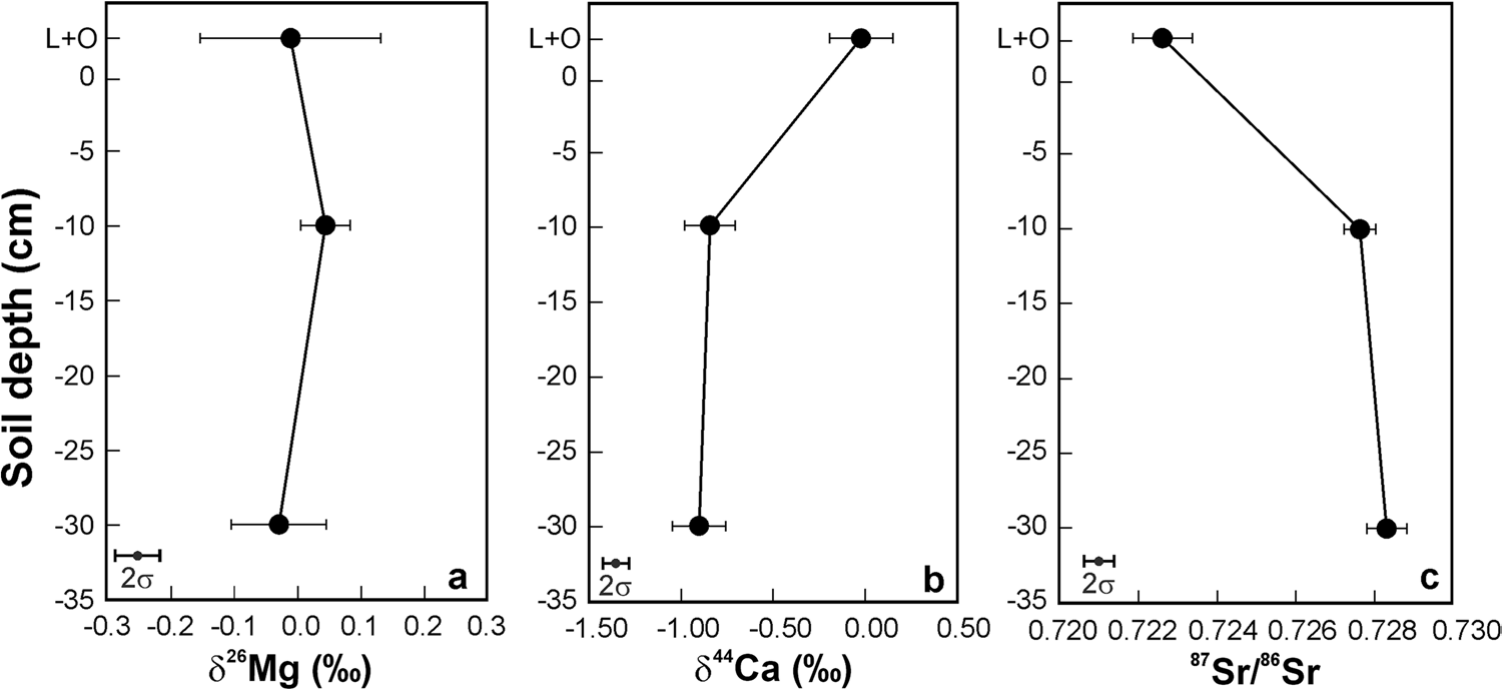
Vertical trends in δ^26^Mg, δ^44^Ca and ^87^Sr/^86^Sr isotope ratios in CER soils. Means and standard errors are given

With an increasing depth, Mg isotope composition of bulk soil did not change outside of the uncertainty of mass spectrometric measurements; δ^44^Ca values decreased, and ^87^Sr/^86^Sr ratios increased (Fig. 3).

Isotope measurements in individual CER samples are in Tab. S1 and S2 and Fig. S4. In Fig. S5, we give two examples of endmember mixing plots according to Capo et al (1998; δ^26^Mg *vs.* Ca/Mg and ^87^Sr/^86^Sr vs. Ca/Sr). In both cases, runoff plotted inside the triangle is defined by throughfall, soil water and bulk soil. In both models, soil water contributed over 90% to base cations in runoff.

### Temporal trends in catchment input–output fluxes

**Fig. 4:**
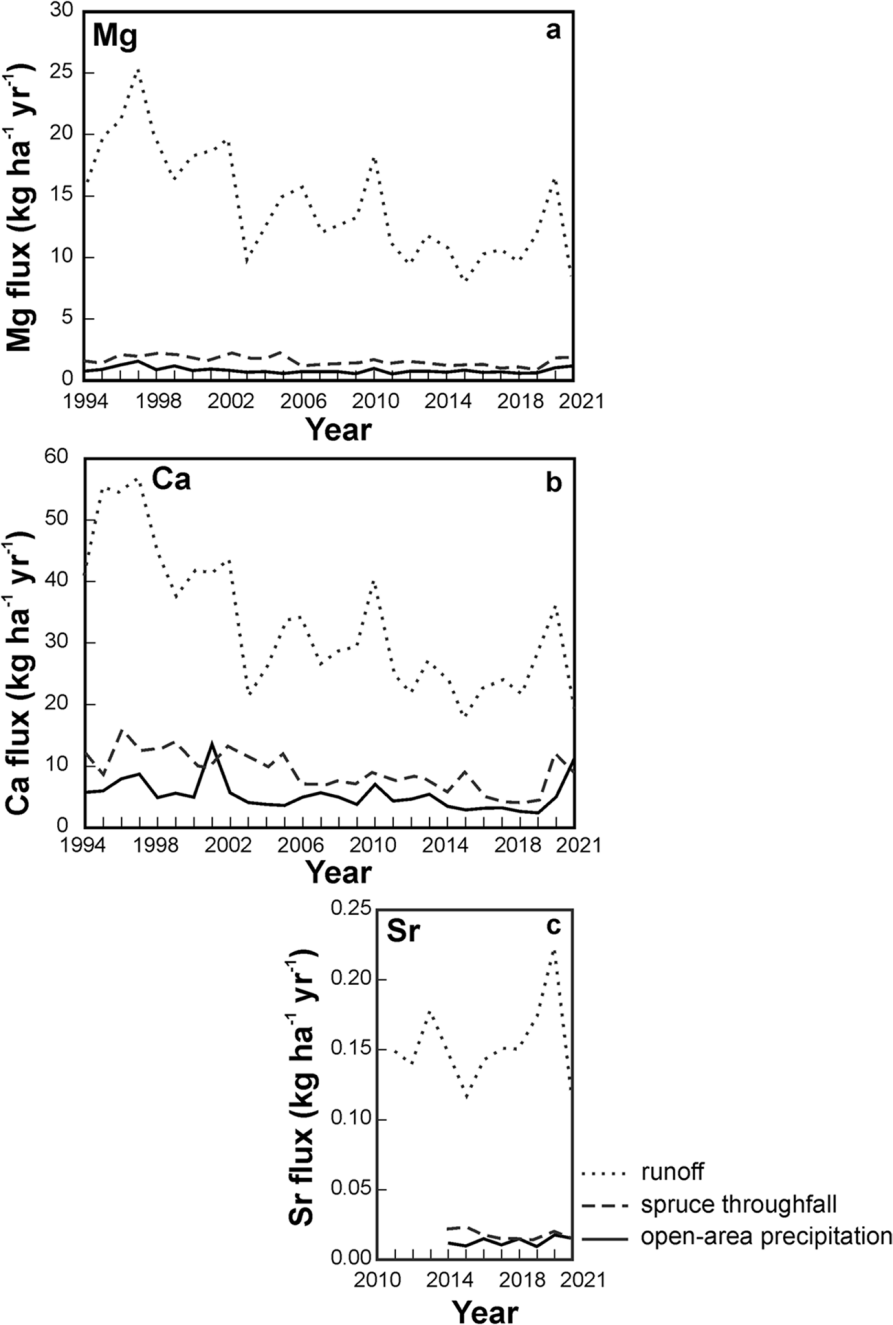
Long-term input–output fluxes of Mg, Ca and Sr at CER

For all the studied base cations, annual fluxes decreased in the order: runoff > > throughfall > open-area precipitation (Fig. 4). This relationship was valid throughout the entire 28-year monitoring period for Mg and Sr. Calcium flux via open-area precipitation was slightly higher than Ca flux via throughfall in 2001 and 2021. Runoff Mg flux between 1994 and 2021 *per* unit area was on average 18 times higher than rainfall Mg input flux. Over the same time period, runoff Ca flux was, on average, six times higher than rainfall Ca flux. The average Sr runoff flux (2014–2021) was 12 times higher than the Sr rainfall flux. Runoff Mg flux was nine times higher than throughfall Mg flux, Ca runoff flux was nine times higher than the throughfall Ca flux, and runoff Sr flux was nine times higher than throughfall Sr flux.

Fig. S6 gives a comparison of average vegetation-type weighted annual input and output fluxes of Mg, Ca and Sr throughout the observation period. Conversion of the measured element fluxes (left bar) from Fig. 4 to total atmospheric deposition (middle bar) using Na concentrations eliminated the effect of scavenging and leaching of base cations by canopy (Kopacek et al. 2016). The correction had only a minor effect on the base cation fluxes. At the catchment scale, throughout the observation period, after the ratio of forested areas and clearings (88:12) was considered and the Na-correction applied, the average annual runoff flux was 15, 5 and 10 times higher for Mg, Ca, and Sr, respectively, compared to the atmospheric input.

A significant decrease in runoff and throughfall fluxes over time was observed for Mg and Ca (*p* < 0.01; Fig. 4a,b). In the case of open-area precipitation, borderline statistical significance was found (*p* = 0.043 for Mg, and *p* = 0.055 for Ca).

### Bedrock chemistry and mineralogy

Mean MgO concentration in bedrock sandstone was approximately eight times lower than MgO concentration in claystone (0.21 *vs.* 1.62 wt. %; Table 2). Mean CaO concentration in bedrock sandstone was approximately three times lower than CaO concentration in claystone (0.15 vs. 0.52 wt. %; Table 2). Concentrations of MgO and CaO in conglomerate was close to those in sandstone. The contents of CO_2_ indicating the potential presence of carbonate were small in all three bedrock types (Table 2), close to the detection limit (0.01 wt. %). According to the geological sketch in Fig. S1, claystone built up a mere 4.7% of the catchment’s area; its occurrence under the deluvial sediments is not known.

**Table 2:** Chemical compositions (wt. % ± SD) of the sandstone, claystone and conglomerate bedrock at the study site

CER	Sandstone	Claystone	Conglomerate
SiO_2_	88.1 ± 4.21	58.2 ± 0.51	92.2
Al_2_O_3_	5.34 ± 1.48	18.7 ± 0.32	3.39
Fe_2_O_3_	1.43 ± 1.27	3.95 ± 0.36	0.97
TiO_2_	0.25 ± 0.07	0.94 ± 0.02	0.08
FeO	0.28 ± 0.10	1.33 ± 0.17	0.30
MgO	0.21 ± 0.10	1.62 ± 0.19	0.20
MnO	0.01 ± 0.01	0.04 ± 0.01	0.01
CaO	0.15 ± 0.06	0.52 ± 0.15	0.20
Na_2_O	0.39 ± 0.21	0.63 ± 0.06	0.37
K_2_O	1.78 ± 0.31	3.80 ± 0.22	0.57
P_2_O_5_	0.04 ± 0.02	0.17 ± 0.14	0.04
CO_2_	< 0.01	0.01	0.01
S_tot_	0.01 ± 0.003	0.01 ± 0.006	0.03
H_2_O	1.55 ± 0.62	6.54 ± 0.13	0.93

PXRD analysis provided semi-quantitative mineralogical composition of CER sandstone and claystone (Table 3). Quartz dominated sandstone (83%), with K-feldspar and albite making up 8 and 6%, respectively. Phyllosilicates constituted less than 4%. Phyllosilicates were the main component of claystone (40%). The applied methodology did not permit to distinguish between mica, illite, and mixed-layered minerals. This group is listed as mica in Table 3. Quartz and kaolinite were the second and third most abundant mineral phases of the studied claystone (< 35 and < 11%). Albite and K-feldspar represented 7 and 4%, respectively.

**Table 3:** PXRD whole-rock analysis of CER sandstone and claystone

Bedrock	Mineral (wt. %)						
	Chlorite	Quartz	Mica	Albite (≤ 10% anorthite component)	K-feldspar	Kaolinite	Anatase
Sandstone		82.8	3.8	5.8	7.7		
Claystone	3	34.5	40	7.1	4.4	10.5	0.5

Thin-section study revealed additional accessory minerals and rock fragments, such as glauconite, ilmenite and zircon in the sandstone (Fig. S7-*I*), and glauconite and lithic clast of magmatic and metamorphic rocks, including rhyolite (Fig. S7-*II, III*) in the conglomerate. Fig. S7-*IV* captured well-preserved silicified relics of benthic foraminifera of the genus *Bathysiphon*.

### Pool size inventory

The aboveground vegetation pool of Mg was only three times larger than the annual runoff flux of Mg (39 *vs.* 14 kg ha^−1^ yr^−1^; Fig. S8). The exchangeable Mg pool was slightly lower in deep soil (40–80 cm below the surface) than in all soil horizons above the depth of 40 cm (31 *vs.* 35 kg ha^−1^ yr^−1^). The aboveground vegetation pool of Ca was 11 times larger than the annual runoff flux of Ca (377 *vs.* 33 kg ha^−1^ yr^−1^; Fig. S9). The exchangeable Ca pool was similar in deep soil (40–80 cm below the surface) and in soil horizons above the depth of 40 cm (149 *vs.* 150 kg ha^−1^ yr^−1^).

## Discussion

### Apparent discrepancy between the isotope and flux approaches

In light of the 5–15 times larger export of Mg, Ca, and Sr via CER runoff, compared to atmospheric input (Fig. S6), we expected isotope similarity between runoff and bedrock rather than between runoff and atmospheric deposition. The opposite was true (Figs. 2 and 4). Below, we will summarize arguments in favor of the isotope hypothesis (“CER runoff mostly contains atmospheric base cations, flux comparisons are misleading”) and arguments in favor of the mass-balance hypothesis (“CER runoff mostly contains geogenic base cations, the isotope fingerprinting is incomplete”). Then, we will suggest further steps to reconcile data in Figs. 2 and 4.

**Fig. 5:**
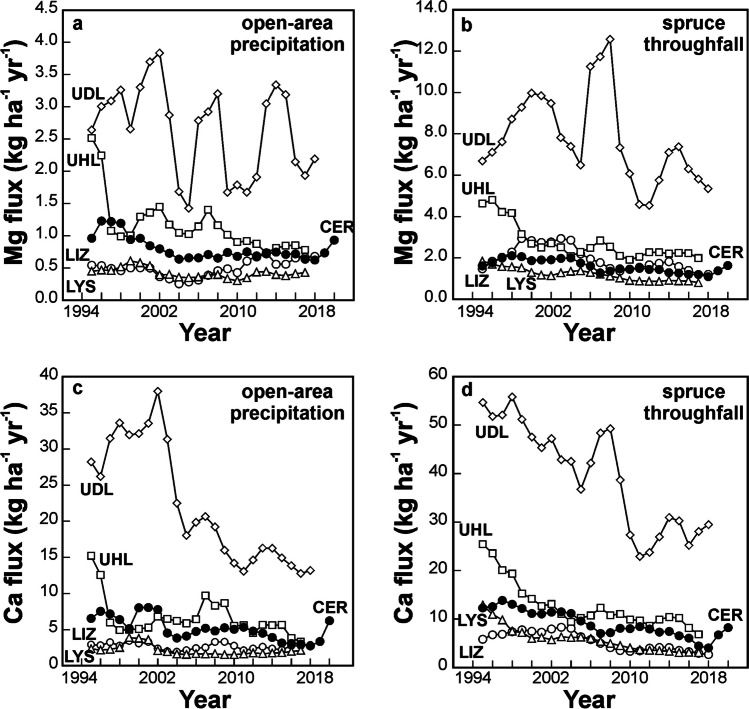
Comparison of magnitude and long-term trends in input Mg, Ca and Sr fluxes in four headwater catchments situated in the Czech Republic differing in their industrial pollution levels (polluted north and unpolluted south, see Fig. 1a for locations). All sites are underlain by base poor crystalline bedrock

CER contrasts with the previously studied Central European catchments that were also underlain by base-poor bedrock (< 2.1 wt. % of MgO and < 1.8 wt. % of CaO) and also exhibited net export of Mg, Ca, and Sr (Fig. 5; Novak et al 2020a, b; Novak et al 2023a,b; for catchment locations see Fig. 1a). Deposition fluxes of the studied base cations at CER were relatively low, closer to deposition fluxes in the rural catchments previously studied in southern and western Czech Republic (LIZ, LYS) than to deposition fluxes in the industrially polluted northern catchments (UDL, UHL). Calcium isotope systematics had been statistically evaluated at four sites. At all these sites, Ca isotope ratios of runoff and bedrock were statistically indistinguishable. Magnesium isotope systematics had been statistically evaluated at two sites. At both, Mg isotope ratios of runoff and bedrock were also statistically indistinguishable. These comparisons highlight the unusual Mg and Ca isotope systematics at CER.

### Arguments in favor of the isotope hypothesis

A large body of literature exists on Mg, Ca and Sr isotope composition of marine carbonates (Tang et al 2008; Li et al 2010; Zhao et al 2022a, b). All three elements exhibit a wide range of isotope ratios. The δ^26^Mg values of marine carbonates span more than 5 ‰ (− 5.3 to 0.0‰; Higgins and Schrag 2015; Liu and Li 2019), while the δ^44^Ca values of recent planktic foraminifera alone span nearly 4‰ (− 2.0 to 1.8 ‰; Gussone et al 2016). The ^87^Sr/^86^Sr ratios of marine carbonates depend on the age. For the 160–26 Ma period corresponding to the formation of the Carpathians, the ^87^Sr/^86^Sr ratios are from 0.706 to 0.708 (Nadaskay et al 2019; McArthur et al. 2020). Isotope signatures of marine carbonates thus span the entire range of CER ecosystem values for δ^44^Ca, and nearly the entire range of CER ecosystem values for δ^26^Mg (*y*-axes in Fig. 2a, b). The ^87^Sr/^86^Sr ratios in the CER ecosystem lie outside the marine carbonate values (y-axis in Fig. 2c). In principle, a missing lithogenic source of base cations, such as sedimentary carbonate layer that was not isotopically analyzed, could shift the combined bedrock isotope signature toward that of runoff. However, it is unlikely that rapid weathering of the unknown carbonate source would simultaneously shift all three investigated isotope ratios (δ^26^Mg, δ^44^Ca and ^87^Sr/^86^Sr) to values practically identical to local atmospheric deposition. Indeed, the improbability of such simultaneous shifts is the strongest argument for a major role of recent precipitation in CER runoff.

Unlike in previously studied Czech catchments, at CER, we were unable to isotopically analyze Mg, Ca and Sr of individual rock-forming and accessory minerals. In previously studied catchments underlain by granite, orthogneiss and paragneiss, we separated up to seven minerals from ground matrix and analyzed them isotopically. We found largely variable δ^26^Mg (-3.3 to 0.0 ‰), δ^44^Ca (-0.6 to 1.0 ‰), and ^87^Sr/^86^Sr (0.714 to 2.05) ratios (Novak et al 2020a, 2020b, 2020c; 2023a, 2023b). These studies confirmed previous reports of large isotope variability between minerals in crystalline rocks (Nezat et al 2007; Farkas et al 2011; Ryu et al 2011; Drouet et al 2015). At CER, it was not possible to separate individual minerals from ground arkose sandstone due to ubiquitous intergrowths, and it was not possible to mechanically separate individual clay minerals from claystone. In Appendix III, we have listed empirical dissolution rate constants of individual minerals present in CER sandstone and claystone. Even without knowledge of reaction surfaces and kinetic calculations, it is obvious that individual phases may, step by step, release into solution Mg, Ca and Sr carrying distinct, mutually contrasting isotope compositions. Yet, we can here use the same argument as we have done in the case of variable isotope composition of basic cations in hypothetical missing carbonate sediments: It remains improbable that differences in weathering regimes of individual CER minerals would simultaneously push δ^26^Mg, δ^44^Ca and ^87^Sr/^86^Sr ratios of dissolved geogenic Mg, Ca and Sr in runoff from the whole-rock values to the almost exact atmospheric deposition values, as seen in Fig. 2.

CER is characterized by steep slopes (an average of 30% according to ArcGIS). Previous biogeochemical research has shown that some atmospherically derived nutrients exhibit shorter mean residence time within steep catchments than at mild-sloping sites (Sueker et al 1999; Michel et al 2000; Novak et al 2004). Hydrological control may cause smaller interaction of atmospherically deposited nutrients with the forest ecosystem at steeper sites, favoring their faster export.

Isotope studies have previously shown that runoff at some of the 14 headwater catchments of the GEOMON network contains atmospheric rather than geogenic S, Pb and Zn (Novak et al 2005; Bohdalkova et al 2014; Andronikov et al 2021). These chemical elements are relatively abundant in crustal rocks, but, at the same time, industrial activities have caused their elevated atmospheric fluxes. Again, rather than suggesting an analogy between the hypothetical high export of atmospheric Mg, Ca and Sr and the isotopically documented predominance of pollutants S, Pb and Zn in catchment runoff in industrial areas, we wish to stress that extremely large reserves of elements in the parental rock do not automatically imply their dominance in runoff.

### Arguments in favor of the mass-balance hypothesis

Hydrochemical mass balances at the small-catchment level are associated with uncertainties stemming, *inter alia,* from a limited number of sampling sites along the slope, discontinuous sampling of runoff, difficult quantification of horizontal deposition, and site-specific interactions between rainfall and tree canopy (Probst et al 1990; Miller et al 1993; Moldan and Cerny 1994; Kohler et al 2015; Schwartz et al 2022). At CER, caution must be exercised given the fact that all deposition samplers were installed at the lowest elevation of the catchment (Fig. 1b). With an increasing elevation, precipitation totals generally increase and may cause additional element input that has not been considered. Havel et al. (1996) found a linear 50 mm increase in annual precipitation total for each additional 100 m elevation in the Ore Mts., northern Czech Republic. Kopacek et al. (2012) showed that at elevations over 900-m the linear increase in precipitation totals is better approximated by an exponential regression and inferred an average 105 mm increase per 100 m elevation for the 300–1550 m a.s.l. span. Havel et al. (1996) monitored concentrations of Mg^2+^ and Ca^2+^ in precipitation along a 500 m elevational gradient in the northern Czech Republic and concluded that the concentrations at individual elevations were statistically indistinguishable. The 324 m difference between the lowest and highest point within CER (Table 1), along with the above previously published findings regarding changing amounts of precipitation but nearly constant Mg and Ca concentrations in rainfall at different elevations, indicate that the uncertainty in catchment level Mg and Ca atmospheric input fluxes at CER is probably low (*cf.,* Pu et al 2023).

Importantly, Oulehle et al. (2017) reported a long-term chloride (Cl^−^) budget for CER. Internal sources of Cl^−^, mainly weathering of the parental rock, are minor. Atmospheric deposition of Cl^−^ is a mixture of marine-aerosol derived and industrial-emission derived Cl^−^. Chloride is believed to be a conservative tracer in forested catchments, moving rapidly through the ecosystem with small to negligible biogeochemical interactions. At CER, decreases in Cl^−^ deposition and export were significantly related, and the Cl^−^ mass budget over the 21-year period was balanced (Oulehle et al 2017). The construction of input–output mass balances at CER is thus reasonably well-constrained.

The one-month sampling interval for chemical and isotopic analysis of CER runoff introduces an uncertainty, especially during the snowmelt period (February–April). While runoff water flux is monitored continuously, the Mg, Ca and Sr isotope composition between runoff samplings was poorly constrained. The δ^26^Mg, δ^44^Ca, and ^87^Sr/^86^Sr ratios of runoff during the peak snowmelt between samplings can be assumed to converge to those of recent precipitation because the portion of runoff that was derived directly from snowpack had been barely in contact with the soil and vegetation and the rates of most biogeochemical processes decrease with decreasing temperatures. Even without continuous isotope analysis of spring runoff, data in Fig. 2 already coincide with isotope signatures of precipitation.

Kram et al. (2012) observed decreasing Mg^2+^ and Ca^2+^ concentrations at higher runoff water fluxes in three small forested catchments in the western Czech Republic. In principle, unmeasured lower concentrations of base cations in runoff between spring samplings may affect the calculation of annual Mg^2+^ and Ca^2+^ export fluxes. Such overestimation of export fluxes of the studied elements, however, will likely be minor (*cf.,* Alewell et al 2004). Overall, we conclude that the 5 to 15 times higher export of the studied base cations from CER relative to the atmospheric input fluxes is too high to be explained by the above-discussed uncertainties in budget calculations. Robustness of the input–output flux estimates is the strongest argument for the predominantly geogenic origin of runoff Mg, Ca and Sr.

Two distinct patterns seen in the CER data (Figs. 2 and 4) *do not* contribute to testing the geogenic *vs.* atmospheric runoff generation hypotheses: (i) decreasing Mg and Ca runoff fluxes in Fig. 4 are not a result of predominating atmospheric origin of these elements in runoff in an era of easing air pollution. The runoff flux decrease is instead a consequence of retreating acidification. Declining concentrations of strong acids in soil solutions (mostly H_2_SO_4_ of atmospheric origin) result in lower leaching of base cations from the soil cation-exchanger (*cf.,* Heliwell et al 2014; Garmo et al. 2014). The input–output time-series in Fig. 4a,b have different slope. Changes in the exchangeable Mg and Ca soil pool may involve both geogenic and atmospheric base cation sources, and therefore, changing export fluxes per se do not carry information in atmospheric *vs.* geogenic runoff origin; (ii) the Mg and Ca isotope composition of xylem as the main plant reservoir plots between the isotope signatures of throughfall and soil (dotted lines in Fig. 2a,b). Hence, simple mixing between soil-derived and atmospheric Mg and Ca may provide a sufficient explanation of xylem isotope ratios. At the same time, it is well established that assimilation by trees prefers isotopically heavy Mg and isotopically light Ca (Page et al 2008; Cobert et al 2011; Hindshaw et al 2013; van der Heijden et al 2015; Kimmig et al 2018). The spruce stands at CER are relatively young ( ≤ 70 yrs), and tree biomass continues to build up. The residual isotopically light Mg and isotopically heavy Ca may shift the isotope signatures of runoff in the direction that has actually been observed (Fig. 2). It is therefore impossible to separate mixing and biological fractionation in Fig. 2. Predominance of residual Mg and Ca in runoff following isotope-selective partial uptake of these elements by plants cannot be taken as an exclusive explanation of isotope patterns in runoff (Fig. 2a,b).

Kinetic-limited chemical erosion that likely predominates at CER over supply-limited erosion (Riebe et al 2004) may have a complex effect on the provenance of base cations in runoff: limited accumulation of soils on the steep slopes results in limited capture of atmospheric inputs by surface soil horizons, and relatively fast export of partly weathered regolith rich in minerals stable under environmental conditions exposes unweathered substrate rich also in unstable minerals. The first phenomenon may enrich runoff in atmospheric base cations, the second phenomenon preferentially supplies stream water with base cations from reactive mineral phases.

### Search for the unknown Mg, Ca and Sr source

Based on the abundances of individual minerals (Table 3) and solid phase stoichiometry, mica combined with illite-group phyllosilicates and plagioclase are the main potential Ca sources in CER claystone. Mica combined with illite-group phyllosilicates and chlorite are the main Mg sources in CER claystone. In alkaline-earth elements poor sandstone, mica, illite-group minerals and plagioclase are the main Ca source, while mica and illite-group minerals are the main Mg source. All these Mg and Ca sources contributed to the whole rock isotope ratios in Fig. 2. If Mg and Ca in silicate analyses in Table 2 are attributed to individual minerals, starting with carbonates expressed as CO_2_, the non-carbonate phases still contain 96.9 to 99.7% of all present Mg and Ca. In other words, we found negligible amounts of an easily soluble (Ca, Mg) CO_3_ phase in the sampled rocks.

Flysch sediments, or turbidites, forming CER parental rock resulted from a combination of fluidal and sediment gravity flow. They were formed as underwater avalanches of uncemented clastic sediments that slid down the steep slope of the continental shelf edge into the deep ocean. Their lithology is highly variable but rarely includes carbonate material. Fig. S10 depicts the lithology of two drill cores (453 and 20705 m deep) which are located at Stare Hamry, 4 km east of CER. These profiles illustrate (i) the large spatial variability of the lithology and (ii) absence of carbonate strata. Despite a mere 30 m distance between the two drill cores, rapid changes in rock types in the shorter core (sandstone, shale, claystone) contrast with the monotonous stratigraphy of the longer core (mainly sandstone, only one layer of claystone intercalations). Neither of these drill cores contains carbonate-rich facies, such as marlstones.

Mencik et al. (1983), Golonka and Picha (2006) and Stranik et al. (2021) published a detailed lithology of the older Godula and younger Istebna Formation. Within the 2900–3100 m thick Godula Formation, the claystones were carbonate-free, but a limited occurrence of calcareous sandstones was reported. Within the 1000–1200-m-thick Istebna Formation, arkose sandstones did not contain carbonate. In contrast, Jurassic and Devonian carbonate cobbles were occasionally present in conglomerates. According to Adamova (1986), dark claystone in the Silesian Unit may also contain subordinated amounts of carbonates (calcite, dolomite and siderite). While we were unable to identify any easily weathering carbonate rocks at CER, their occurrence in the broader region cannot be ruled out. On a smaller scale, small amounts of remobilized carbonate may have remained in the Silesian Unit after silicification of the carbonate fossils, such as foraminifera in Fig. S7-*IV*.

We suggest that there may be an analogy between carbonate-derived basic cations in runoff from catchments underlain by heterogeneous flysch sediments and the predominance of carbonate-derived basic cations in rivers draining silicate catchments (Moore et al 2013; Fantle and Tipper 2014; Jacobson et al 2015; Xu et al 2022). Small volumes of carbonate veinlets were shown to supply a large proportion of riverine Ca^2+^ in granitoid terranes. In the Carpathian Flysch sediments, we do not expect hydrothermal calcite veinlets, but subordinated amounts of carbonate components of conglomerates, marlstones and calcareous sandstones may contribute to reconciling CER data in Fig. 2 and 4.

Fig. S11 outlines a hypothetical isotope scenario of silicate and carbonate dissolution and its possible effect on δ^26^Mg and ^87^Sr/^86^Sr ratios of runoff (*cf.*, Zhao et al 2022a). The scenario assumes a global average δ^26^Mg and ^87^Sr/^86^Sr isotope ratios of silicate and marine carbonate rocks for the studied area. For both Mg and Sr, the silicate mixing endmember is isotopically heavier, and the carbonate mixing endmember is isotopically lighter than Mg and Sr in CER runoff. Such mixing, if empirically confirmed, would corroborate the above-discussed mass balance hypothesis stating the dominance of geogenic sources of base cations in CER runoff. Global mean δ^44^Ca values are similar for silicate and carbonate rocks (Blättler and Higgins 2017), and hence, Ca isotopes cannot be used for a model analogous to Fig. S11.

### Relationship between Sr isotope ratios in runoff and vertical soil profiles

Generally, a linear relationship between 1/concentration and isotope ratios in stream water (Fig. S3d) suggests mixing between two sources of an element (*e.g.,* Zhao et al 2022b). Comparison between Figs. S3d and 3c could, in principle, indicate whether Sr in runoff in a wet month converges to a shallow or a deep soil horizon as its potential source. Unfortunately, the two low Sr-concentration, low-^87^Sr/^86^Sr runoff samples come from hydrologically contrasting times of the year (wet April and dry September). Both these runoff samples converge to the topmost soil horizon having the lowest ^87^Sr/^86^Sr ratio, which, however, remains strikingly different from the runoff Sr isotope signature (0.7225 *vs.* 0.714–0.715). Longer time-series of isotope data would be needed to more rigorously address possible within-soil sources of runoff Sr.

## Conclusions

In a small forested catchment underlain by turbidite sediments, we found nearly identical values for atmospheric deposition and runoff in each of the δ^26^Mg, δ^44^Ca and ^87^Sr/^86^Sr isotope systems. In contrast, each of the Mg, Ca and Sr isotope ratios was significantly different between bedrock and runoff. This was counter-intuitive since long-term hydrogeochemical monitoring of catchment fluxes showed a large net export of each of the three studied base cations via runoff. Over the last 28 hydrological years, the average Mg input via atmospheric deposition was 15 times lower than Mg runoff flux, Ca deposition was 5 times lower than Ca runoff, and Sr deposition was 10 times lower, compared to its hydrological export. Bedrock sandstone and claystone contained only traces of easily dissolving carbonates. We explored the possibility of sizeable export of legacy pollutants Mg, Ca, and Sr that would have been deposited in the high-pollution years 1950–1995. Our mass-budget monitoring started in 1994, and already then, atmospheric Mg, Ca and Sr inputs were small relative to their runoff fluxes. Therefore, the remobilization of legacy pollution from power plant dust temporarily stored in the soil and biomass could not explain the large net export of base cations during our study. We discussed possible sources of uncertainty in both isotope and non-isotope flux data. Uncertainties in catchment-level mass budgets caused by placing precipitation collectors only at the foot of the steep catchment, unknown upslope gradients in base cation concentrations in atmospheric deposition, and discontinuous runoff sampling for concentration and isotope analysis were probably too low to minimize the difference between export and deposition flux of Mg, Ca and Sr. An unknown deeper source of carbonate Ca, Mg and Sr that has not been analyzed isotopically may help to reconcile the apparent isotope–flux discrepancy. Still, it appears to be unlikely that the isotope composition of unknown carbonates would shift the isotope compositions of all three studied elements in runoff almost exactly to those of present-day atmospheric deposition.

### Electronic supplementary material

Below is the link to the electronic supplementary material.

Supplementary file1 Fig. S1. Geological sketch of the CER catchment (Mencik and Pesl 1995). (TIF 35872 KB)

Supplementary file2 Fig. S2. Annual S and N_r_ depositions at CER (Oulehle *et* *al* 2021). (TIF 8531 KB)

Supplementary file3 Fig. S3. Monthly time-series of δ^26^Mg, δ^44^Ca and ^87^Sr/^86^Sr ratios in catchment inputs and output (*a*-*c*). Negative correlation between 1/[Sr] and ^87^Sr/^86^Sr in runoff (*d*). No such relationships were observed for Ca and Mg. (TIF 8528 KB)

Supplementary file4 Fig. S4. Individual isotope measurements used in Fig. 2. (TIF 8528 KB)

Supplementary file5 Fig. S5. Mixing plots according to Capo et al (1998) using throughfall, soil water and bulk soil as the mixing endmembers. In both models, soil water appeared to contribute more than 90 % of the studied base cations to runoff. These models provide qualitative rather than quantitative estimates because of the known non-conservative behavior of some of the tracers and incompatible matrix in liquid vs. solid concentration measurements. For more detailed discussion of the limitations of the endmember mixing plots *see*, *e*.*g*., Novak *et* *al* (2020c). (TIF 8528 KB)

Supplementary file6 Fig. S6. Comparison of average annual input and output fluxes of Mg, Ca, and Sr throughout the observation period. Conversion of the measured element fluxes (left bar) from Fig. 4 to total atmospheric deposition (middle bar) eliminated the effect of scavenging and leaching of base cations by canopy (Kopacek et al., 2016). (TIF 8528 KB)

Supplementary file7 Fig. S7. Mic
rophotographs of thin sections from CER bedrock. *I* – coarse-grained arcosic sandstone with glauconite (parallel nicols); *II* – lithic conglomeratic sandstone to fine-grained conglomerate with clast of glauconitic sandstone, quartzite and mylonitized quartz grains and clastic white mica in the matrix (crossed nicols); *III* – coarse-grained lithic sandstone to fine-grained conglomeratic sandstone rich in lithic clast of magmatic and metamorphic rocks. Small pebble of rhyolite crystalloclastic tuff (parallel nicols); *IV* – laminated dark gray-green aleuropelite with quartz-dominated silty admixture and preserved silicified relics of benthic foraminifera of the genus *Bathysiphon* (parallel nicols). (TIF 35872 KB)

Supplementary file8 Fig. S8. Mg pool size inventory in the CER catchment. (TIF 8530 KB)

Supplementary file9 Fig. S9. Ca pool size inventory in the CER catchment. (TIF 8528 KB)

Supplementary file10 Fig. S10. Schematic representation of the lithology of two drill cores from Stare Hamry, 4 km east of CER. ‘Geofond’ archive of the Czech Geological Survey, Prague. (TIF 8528 KB)

Supplementary file11 Fig. S11. Hypothetical mixing of silicate and carbonate sources of Mg (*a*) and Sr (*b*) plotted together with CER runoff. The graphs use global mean δ^26^Mg and ^87^Sr/^86^Sr isotope ratios for each rock type, except for Sr in silicate sediments for which the global range of values is extremely broad, depending on age and Rb contents (0.705-0.750; Faure and Mensing 2004). Silicate sediment in panel (*b*) is represented by CER claystone (solid circle) and Central European marine sediments of a similar age (80-60 My *BP*; Nadaskay *et* *al* 2019; dotted circle). In the case of both elements, CER runoff plots between the higher isotope ratios of silicates and lower isotope ratios of carbonates, and, in principle, can be derived from dissolution of these two geogenic sources. Mean isotope signatures of mixing endmembers according to Gussone *et* *al* (2016), Teng (2017) and Zhao (2022a) and references therein. Isotope ratios in CER throughfall are marked by a horizontal dashed line. (TIF 8528 KB)

Supplementary file12 (DOCX 24 KB)

Supplementary file13 (DOCX 22 KB)

Supplementary file14 (DOCX 15 KB)

## Data Availability

The entire dataset used is provided in Supplementary Information. Reasonable requests for additional data would be addressed by the corresponding author.

## Appendix 1. Lithology and mineralogy of the Godula and Istebna Formations

The Silesian Unit builds an outer part of a synorogenic accretionary prism of the Carpathians. A wedge-shaped structure was formed during the closure of oceanic Tethys domains and the subsequent collision of the central Carpathian plate with the Brunovistulian foreland. The Mesosoic pre-flysch, syn-rift, and syn-orogenic flysch units of the Outer Western Carpathians were folded, thrusted on the foreland and uplifted to the present-day mountain-range position. The older Godula Formation is composed of rhythmic alteration of sandstones and claystones/siltstones, with conglomerate intercalations. The younger Istebna Formation is composed of slope-apron and submarine-fan sandstones and arkosic sandstones with conglomerate intercalations. The flysch deposits are intercalated by dark marlstones and claystones and dark shales. The bedrock at CER is formed by a brachynticline of flysch sediments of the Istebna Fomation, whereas rocks of the Godula Formation crop out north and east of CER. The lithology of the psammitic members of both formations is similar. Istebna sandstones are mostly coarser-grained in comparison with the sandstones of the lower segment of the Godula Formation. The Istebna Formation sandstones contain more feldspars and conglomerate interbeds. The presence of pebbles and heavy minerals (assemblages dominated by garnet and zircon) indicates that the dominant source of basin turbidites originated in the crystalline basement of the Silesian cordillera. In addition to quartz pebbles and stable silica-rich clast (cherts, muscovite–biotite quartzites), polymict conglomerates contain frequent unstable components, such as orthogneisses, felsic volcanics (tuffs and spherulithic lavas), aplitic granitoids, and large feldspar clast. Recycled glauconitic sandstones are present scarcely. Siderite is the prevailing cementing mineral in psamites with low carbonate content. Godula Formation sandstones are richer in diagenetic glauconite than Istebna Formation. Dark greenish to dark grey/brown aleuropelites/shales rich in Fe oxides (hematite, limonite) and siderite contain sulfides (pyrite) and carbonates only as accessory phases. Pelitic and aleuritic members of both formations are similar. In addition to dominant clay minerals, micas, chlorite, and glauconite, these members contain a silty siliciclastic admixture (quartz, feldspar). Organic matter contents (1–8%) tend to be higher in aleuropelites and shales of the Istebna Formation.

## Appendix 2. Isotope mixing models for Mg, Ca and Sr in forest ecosystems

To constrain the sources and relative contributions of Mg, Ca and Sr into local ecosystem compartments, we calculated theoretical mixing trends for selected ‘end-members’ (see perimeters of grey shapes in Fig. S5). The mixing trends for δ^26^Mg values, δ^44^Ca values, ^87^Sr/^86^Sr isotope ratios, Ca/Mg and Ca/Sr concentration ratios were constructed considering the following end-members: (i) bulk soil, (ii) throughfall, and (iii) soil water. The presented mixing trends were modelled for CER using the elemental and isotope compositions for the above potential end-members given in Tab. S1 and Tab. S2.

For example, the corresponding two-end member mixing trends for δ^26^Mg and ^87^Sr/^86^Sr were calculated based on an isotope mass balance equation below for the mixing of (i) bulk soil (SOIL) and (ii) throughfall (TF) derived Mg sources in a theoretical mixture:

$${\delta Mg}_{MIX}=\left({F}_{SOIL}{\delta }_{SOIL}{C}_{SOIL}+{F}_{TF}{\delta }_{TF}{C}_{TF}\right)/\left({F}_{SOIL}{C}_{SOIL}+{F}_{TF}{C}_{TF}\right)$$

where *δMg*_*MIX*_ represents the Mg isotope composition of the ‘mixed’ (soil + throughfall) reservoir, and the parameters *F*_*SOIL*_*, δ*_*SOIL*_ and *C*_*SOIL*_ represent, respectively, the fraction (F, from 0 to 1), the isotope signature and the Mg concentration of the bulk soil (SOIL) reservoir. An identical coding but with a subscript ‘*TF*’ was used for the Mg sourced from local throughfall.

A similar approach was used for the calculation of Sr isotope composition (^87^Sr/^86^Sr) of the mixture, labelled below as Sr**|**Sr_MIX_, based on the following equation:

$$\left.Sr\right|{Sr}_{MIX}=\left({F}_{SOIL}\left.Sr\right|{Sr}_{SOIL}{C}_{SOIL}+{F}_{TF}\left.Sr\right|{Sr}_{TF}{C}_{TF}\right)/\left({F}_{SOIL}{C}_{SOIL}+{F}_{TF}{C}_{TF}\right)$$

Finally, the Ca/Mg ratio of the mixture was calculated using simple elemental mass balance equations (see below), calculated separately for Ca and Sr sources or end-members:

$${Ca}_{MIX}=\left({F}_{SOIL}{C}_{SOIL}+{F}_{TF}{C}_{TF}\right);{Mg}_{MIX}=\left({F}_{SOIL}{C}_{SOIL}+{F}_{TF}{C}_{TF}\right)$$

where *F*_*SOIL*_ and *C*_*SOIL*_ represent, respectively, the fraction (F, from 0 to 1) and the concentration of Ca derived from the bulk soil (SOIL) reservoir; the fraction of Ca derived from precipitation can be calculated as ${F}_{TF}=1-{F}_{SOIL}$.

The concentration of Sr in the mixture (Sr_MIX_) was calculated using an identical approach, and the corresponding Ca/Sr ratio of the mixture was then calculated by dividing Ca_MIX_ by Sr_MIX_.

The concentration of Mg in the mixture (Mg_MIX_) was calculated using on identical approach, and the corresponding Ca/Mg ratio of the mixture was then calculated by dividing Ca_MIX_ by Mg_MIX_.

## Appendix 3. Relative rates of dissolution of CER bedrock minerals

Rates of dissolution of minerals in the studied sandstones and claystones depend on rate constants (k_*d*_), reaction surfaces of the minerals, and the rate of water percolation through the rock complexes.

**Table:**

		Minimum dissolution rate constant *k*_*d*_	Maximum dissolution rate constant *k*_*d*_	References
		mol m^−2^ s^−1^		
Kaolinite	H_4_Al_2_Si_2_O_9_	7.9 10^–15^	5.0 10^–12^	Huertas et al. (1999), Ganor et al. (1995)
Illite	K_0.65_ Al_2_ (Al,Mg,Fe)_0.65_ Si_3_._35_O_10_OH_2_	1.0 10^–14^	1.0 10^–13^	Kohler et al. (2003)
Quartz	SiO_2_		1.70 10^–14^	Dove (1994)
K-feldspar	KAlSi_3_O_8_	1.6 10^–12^	6.3 10^–11^	Siegel and Pfannkuch (1984), Swoboda-Colberg and Drever (1993)
Mica	H_2_KAl_3_(SiO_4_)_3_	3.2 10^–12^	1.6 10^–10^	Kalinowski and Schweda (1996)
Biotite	K(Mg,Fe^2+^)_3_[(OH,F)_2_(Al,Fe^3+^)Si_3_O_10_	2.0 10^–13^	1.0 10^–9^	Lucas et al. (2017), Wolff-Boenisch et al. (2006)
Chlorite	(Mg,Fe)_5_Al(Si_3_Al)O_10_(OH)_8_	3.2 10^–12^	5.0 10^–10^	Brandt et al. (2003)
Albite	NaAl_2_Si_3_O_8_	-11.9	-10	Knauss and Wolery (1986), Welch and Ullman (1996)
Anorthite	CaAl_2_Si_2_O_8_	-10.8	-8.9	Amrhein and Suarez (1992), Oelkers and Schott (1995)
Albite An 10	Na_x_Ca_(1-x)_Al_2-x_Si_2+x_O_8_	2.7 10^–12^	2.2 10^–10^	Calculated: 0.9 k(Ab) + 0.1 k(An)

The mass balance of the weathering is

$$\mathrm{A }=\mathrm{ D }+\mathrm{ R }-\mathrm{ Q},$$

where A is an accumulation of the elements in the rock, D is the atmospheric deposition of the elements, R is the rate of dissolution of the elements from the rock, and Q is the removal of elements by percolating water. Units are mol. m^−3^ s^−1^.

Assuming that the reactive surface *s* of albite (An10) in sandstone is 10^2^ m^−2^ m^−3^, then the rate of dissolution *R* = k_*d*_. *s* is 1.9 10^–10^ to 1.0 10^–8^ mol m^−3^ s^−1^. The rate of release of Ca from albite (An10) in sandstone is R * 0.1, hence 1.9 10^–11^ to 1.0 10^–9^ mol m^−3^ s^−1^, or 8 10^–8^ to 4 10^–7^ g m^−3^ s^−1^.

The content of albite (An10) in sandstone and claystone is similar, 5.8 and 7.1 wt. % (Table 3 in the main text). Unknown is the difference in the reacting surface area. This will certainly be much larger in claystone than in sandstone. If we assume a 50-fold difference, the rate of Ca release from claystone is 4 10^–6^ to 2.0 10^–5^ g m^−3^ s^−1^.

The calculation of the dissolution rate of Mg is even more uncertain. Let us assume that Mg contribution from chlorite is negligible. The main Mg sources are then illite and biotite. In the X-ray analysis, illite is included in muscovite. Biotite was identified in thin sections. The dissolution rate constant for illite is from 1.0 10^–14^ to 1.0 10^–13^ mol m^−2^ s^−1^. The dissolution rate constant for biotite is from 2.0 10^–13^ to 1.0 10^–9^ mol m^−2^ s^−1^. The higher rate constants for dissolution of biotite indicate that the main source of Mg at the weathering front is biotite rather than illite. The average MgO content in sandstone is 0.21 wt. %. The average MgO content in claystone is 1.6 wt. % (Table 1 in the main text). Assuming that the active surface of biotite is the same in sandstone and in claystone, the rate of dissolution of Mg from claystone will be faster by a factor of 7.6.

## References

[CR1] Åberg G, Jacks G, Wickman T, Hamilton PJ (1990). Strontium isotopes in trees as an indicator for calcium availability. Catena.

[CR2] Adamova M (1986). Geochemical evaluation of the sediments of the Silesian unit. J Geol Sci Geol.

[CR3] Alewell C, Lischeid G, Hell U, Manderscheid B (2004). High temporal resolution of ion fluxes in semi-natural ecosystems–gain of information or waste of resources?. Biogeochemistry.

[CR4] Amrhein C, Suarez DL (1992). Some factors affecting the dissolution kinetics of anorthite at 25°C. Geochim Cosmochim Acta.

[CR5] Andronikov AV, Novak M, Oulehle F, Chrastny V, Sebek O, Andronikova IE, Stepanova M, Sipkova A, Hruska J, Myska O, Chuman T, Veselovsky F, Curik J, Prechova E, Komarek A (2021). Catchment runoff in industrial areas exports legacy pollutant zinc from the topsoil rather than geogenic Zn. Environ Sci Technol.

[CR6] Antonelli MA, Yakymchuk C, Schauble EA, Foden J, Janousek V, Moyen JF, Hoffmann J, Moynier F, Bachmann O (2023). Granite petrogenesis and the δ^44^Ca of continental crust. Earth Planet Sci Lett.

[CR7] Basdedios N, Wu Y, Wilcke W (2023). Magnesium isotope ratios reflect the size and source of Mg loss along a glacial retreat chronosequence. ACS Earth Space Chem.

[CR8] Bedel L, Poszwa A, van der Heijden G, Legout A, Aquilina L, Ranger J (2016). Unexpected calcium sources in deep soil layers in low-fertility forest soils identified by strontium isotopes (Lorraine plateau, eastern France). Geoderma.

[CR9] Belanger N, Holmden C (2010). Influence of landscape on the apportionment of Ca nutrition in a Boreal Shield forest of Saskatchewan (Canada) using ^87^Sr/^86^Sr as a tracer. Can J Soil Sci.

[CR10] Black JR, Epstein E, Rains WD, Yin Q-Z, Casey WH (2008). Magnesium-isotope fractionation during plant growth. Environ Sci Technol.

[CR11] Blättler CL, Higgins JA (2017). Testing Urey's carbonate–silicate cycle using the calcium isotopic composition of sedimentary carbonates. Earth Planet Sci Lett.

[CR12] Bohdalkova L, Novak M, Stepanova M, Fottova D, Chrastny V, Mikova J, Kubena AA (2014). The fate of atmospherically derived Pb in Central European catchments: insights from spatial and temporal pollution gradients and Pb isotope ratios. Environ Sci Technol.

[CR13] Bolou-Bi EB, Vigier N, Poszwa A, Boudot J-P, Dambrine E (2012). Effects of biogeochemical processes on magnesium isotope variations in a forested catchment in the Vosges Mountains (France). Geochim Cosmochim Acta.

[CR14] Bouchez J, von Blanckenburg F (2021). The role of vegetation in setting strontium stable isotope ratios in the Critical Zone. Am J Sci.

[CR15] Brandt F, Bosbach D, Krawczyk-Barsch E, Arnold T, Bernhard G (2003). Chlorite dissolution in the acid pH-range: a combined microscopic and macroscopic approach. Geochim Cosmochim Acta.

[CR16] Brantley SL, White TS, White AF, Sparks D, Richter D, Pregitzer K, Derry L, Chorover J, Chadwick O, April L, Anderson S, Amundson R (2005) Frontiers in exploration of the critical zone. Workshop on Frontiers in Exploration of the critical Zone 24-26 Oct 2005, Report of a Workshop, National Science Foundation, Newark (Delaware)

[CR17] Brazier J-M, Schmitt A-D, Gangloff S, Pelt E, Chabaux F, Tertre E (2019). Calcium isotopic fractionation during adsorption onto and desorption from soil phyllosilicates (kaolinite, montmorillonite and muscovite). Geochim Cosmochim Acta.

[CR18] Brenot A, Baran N, Petelet-Giraud E, Negrel P (2008). Interaction between different water bodies in a small catchment in the Paris basin (Brevilles, France): tracing of multiple Sr sources through Sr isotopes coupled with Mg/Sr and Ca/Sr ratios. Appl Geochem.

[CR19] Brenot A, Cloquet C, Vigier N, Carignan J, France-Lanord C (2008). Magnesium isotope systematics of the lithologically varied Moselle river basin, France. Geochim Cosmochim Acta.

[CR20] Brewer A, Teng FZ, Dethier D (2018). Magnesium isotope fractionation during granite weathering. Chem Geol.

[CR21] Bruker AXS (2014) Topas 5. Karlsruhe, Germany

[CR22] Bruker AXS (2015) Diffrac.Eva, 4.1. Karlsruhe, Germany

[CR23] Bukoski IS, Murphy SF, Birch AL, Barnard HR (2021). Summer runoff generation in foothill catchments of the Colorado Front Range. J Hydrol.

[CR24] Bullen TD, Fitzpatrick JA, White AF, Schulz MS, Vivit DV (2004) Calcium stable isotope evidence for three soil calcium pools at a granitoid chronosequence. In Water–rock Interaction. Proceedings of the eleventh international symposium on water–rock interaction, Vol. 1. Saratoga Springs, New York, pp 813–817

[CR25] Burger A, Lichtscheidl I (2019). Strontium in the environment: review about reactions of plants towards stable and radioactive strontium isotopes. Sci Total Environ.

[CR26] Buzek F, Cejkova B, Hellebrandova L, Jackova I, Lollek V, Lnenickova Z, Matolakova R, Veselovsky F (2017). Isotope composition of NH_3_, NO_x_ and SO_2_ air pollution in the Moravia-Silesian region, Czech Republic. Atmos Pollut Res.

[CR27] Buzek F, Cejkova B, Jackova I, Seibert R, Curik J, Veselovsky F, Petrash DA (2023). Tracking sources of PM_10_ emissions and deposition in the industrial city of Ostrava, Czech Republic: a carbonaceous δ^13^C-based approach. Atmos Environ.

[CR28] Cai D, Henehan MJ, Uhlig D, Von Blanckenburg F (2022). Mg isotope composition of runoff is buffered by the regolith exchangeable pool. Geochim Cosmochim Acta.

[CR29] Capo RC, Stewart BW, Chadwick OA (1998). Strontium isotopes as tracers of ecosystem processes: theory and methods. Geoderma.

[CR30] Cenki-Tok B, Chabaux F, Lemarchand D, Schmitt A-D, Pierret M-C, Viville D, Bagard M-L, Stille P (2009). The impact of water–rock interaction and vegetation on calcium isotope fractionation in soil- and stream waters of a small, forested catchment (the Strengbach case). Geochim Cosmochim Acta.

[CR31] Chadwick OA, Derry LA, Vitousek PM, Huebert BJ, Hedin LO (1999). Changing sources of nutrients during four million years of ecosystem development. Nature.

[CR32] Chapela Lara M, Buss HL, von Strandmann PAE, Schuessler JA, Moore OW (2017). The influence of critical zone processes on the Mg isotope budget in a tropical, highly weathered andesitic catchment. Geochim Cosmochim Acta.

[CR33] Chen BB, Li SL, von Strandmann PAP, Wilson DJ, Zhong J, Ma TT, Sun J, Liu CQ (2023). Behaviour of Sr, Ca, and Mg isotopes under variable hydrological conditions in high-relief large river systems. Geochim Cosmochim Acta.

[CR34] Cobert F, Schmitt A-D, Bourgeade P, Labolle F, Badot P-M, Chabaux F, Stille P (2011). Experimental identification of Ca isotopic fractionations in higher plants. Geochim Cosmochim Acta.

[CR35] Court M, van der Heijden G, Louvat P, Bolou-Bi E, Caro G, Bouchez J, Pollier B, Didier S, Nys C, Saint-Andre L, Legout A (2021). Mg isotope composition in beech forest ecosystems and variations induced by liming: Insights from four experimental sites in Northern France. Biogeochemistry.

[CR36] de Villiers S, Dickson JAD, Ellam RM (2005). The composition of the continental river weathering flux deduced from seawater Mg isotopes. Chem Geol.

[CR37] Demonterova EI, Ivanov AV, Sklyarov EV, Pashkova GV, Klementiev AM, Tyagun ML, Vanin VA, Vologina EG, Yakhnenko AS, Yakhnenko MS, Kozyreva EA (2022). ^87^Sr/^86^Sr of Lake Baikal: evidence for rapid homogenization of water. Appl Geochem.

[CR38] Dove PM (1994). The dissolution kinetics of quartz in sodium chloride solutions at 25 degrees to 300 degrees C. Am J Sci.

[CR39] Drouet T, Herbauts J, Demaiffe D (2005). Long-term records of strontium isotopic composition in tree rings suggest changes in forest calcium sources in the early 20th century. Glob Chang Biol.

[CR40] Drouet T, Herbauts J, Demaiffe D (2015). Influence of deep soil horizons on Ca nutrition of forest stands along a loessic soil sequence. Plant Soil.

[CR41] Erban Kochergina YV, Erban V, Hora JM (2022). Sample preparation and chromatographic separation for Sr, Nd, and Pb isotope analysis in geological, environmental, and archaeological samples. J Geosci-Czech.

[CR42] Fahad ZA, Bolou-Bi EB, Köhler SJ, Finlay RD, Mahmood S (2016). Fractionation and assimilation of Mg isotopes by fungi is species dependent. Env Microbiol Rep.

[CR43] Fan B, Yang X, Jiang K, Zhao Z (2023). Processes controlling the Mg isotope behavior during granite weathering. J Asian Earth Sci.

[CR44] Fantle MS, Tipper ET (2014). Calcium isotopes in the global biogeochemical Ca cycle: Implications for development of a Ca isotope proxy. Earth-Sci Rev.

[CR45] Farkas J, Dejeant A, Novak M, Jacobsen SB (2011). Calcium isotope constraints on the uptake and sources of Ca^2+^ in a base-poor forest: a new concept of combining stable (δ^44/42^Ca) and radiogenic (εCa) signals. Geochim Cosmochim Acta.

[CR46] Faure G, Mensing TM (2004) Isotopes: principles and applications. 3rd. John Wiley & Sons Inc., p 546

[CR47] Fottova D, Skorepova I (1998). Changes in mass element fluxes and their importance for critical loads: GEOMON network, Czech Republic. Water Air Soil Pollut.

[CR48] Fries DM, James RH, Dessert C, Bouchez J, Beaumais A, Pearce CR (2019). The response of Li and Mg isotopes to rain events in a highly-weathered catchment. Chem Geol.

[CR49] Ganor J, Mogollon JL, Lasaga AC (1995). The effect of pH on kaolinite dissolution rates and on activation energy. Geochim Cosmochim Acta.

[CR50] Garmo ØA, Skjelkvale BL, de Wit HA, Colombo L, Curtis C, Folster J, Hoffmann A, Hruška J, Høgasen T, Jeffries DS, Keller WB, Kram P, Majer V, Monteith DT, Paterson AM, Rogora M, Rzychon D, Steingruber S, Stoddard JL, Vuorenamaa J, Worsztynowicz A (2014). Trends in surface water chemistry in acidified areas in Europe and North America from 1990 to 2008. Water Air Soil Pollut..

[CR51] Golonka J, Waskowska A, Slaczka (2019) The western outer Carpathians: origin and evolution. Journal of Applied and Regional Geology/Z Dtsch Ges Geowiss 170(3/4):229

[CR52] Golonka J, Picha FJ (eds) (2006) The Carpathians and their foreland: Geology and hydrocarbon resources. AAPG Memoir 84, vol 84. AAPG, U.S.A., 835 p. ISBN print: 0891813651. ISBN electronic: 9781629810379. 10.1306/M84985

[CR53] Guo B, Zhu X, Dong A, Yan B, Shi G, Zhao Z (2019). Mg isotopic systematics and geochemical applications: a critical review. J Asian Earth Sci.

[CR54] Gussone N, Schmitt A-D, Heuser A, Wombacher F, Dietzel M, Tipper E, Schiller M (Eds) (2016) Calcium stable isotope geochemistry. Springer-Verlag, Berlin. 10.1007/978-3-540-68953-9

[CR55] Havel M, Krejci R and Cerny J (1996) Decrease in acid deposition in the Ore Mts. Czech Geological Survey, Final Project Report 6102, Prague, pp 176

[CR56] Heliwell RC, Wright RF, Jackson-Blake LA, Ferrier RC, Aherne J, Cosby BJ, Evans CD, Forsius M, Hruška J, Jenkins A, Kram P, Kopacek J, Majer V, Moldan F, Posch M, Potts JM, Rogora M, Schopp W (2014). Assessing recovery from acidification of European surface waters in the year 2010: evaluation of projections made with the MAGIC model in 1995. Environ Sci Technol.

[CR57] Higgins JA, Schrag DP (2015). The Mg isotopic composition of Cenozoic seawater–evidence for a link between Mg-clays, seawater Mg/Ca, and climate. Earth Planet Sci Lett.

[CR58] Hindshaw RS, Reynolds BC, Wiederhold JG, Kiczka M, Kretzschmar R, Bourdon B (2013). Calcium isotope fractionation in alpine plants. Biogeochemistry.

[CR59] Hindshaw RS, Tosca R, Tosca NJ, Tipper ET (2020). Experimental constraints on Mg isotope fractionation during clay formation: implications for the global biogeochemical cycle of Mg. Earth Planet Sci Lett.

[CR60] Holmden C, Belanger N (2010). Ca isotope cycling in a forested ecosystem. Geochim Cosmochim Acta.

[CR61] Holmden C (2005) Measurement of δ^44^Ca using a ^43^Ca-^42^Ca double-spike TIMS technique. In: Summary of Investigations 2003, volume 1, Saskatchewan Geological Survey, Saskatchewan Industry and Resources, Miscellaneous Report 2005–1,CD-ROM, Paper A-4 7p

[CR62] Hruška J, Moldan F, Kram P (2002). Recovery from acidification in central Europe–observed and predicted changes of soil and streamwater chemistry in the Lysina catchment, Czech Republic. Environ Pollut.

[CR63] Hruška J, Oulehle F, Chuman T, Kolar T, Rybnicek M, Trnka M, McDowell WH (2023). Forest growth responds more to air pollution than soil acidification. Plos One.

[CR64] Huertas FJ, Chou L, Wollast R (1999). Mechanism of kaolinite dissolution at room temperature and pressure. Part II. Kinetic study. Geochim Cosmochim Acta.

[CR65] Hunova I, Brabec M, Maly M, Valerianova A (2018). Revisiting fog as an important constituent of the atmosphere. Sci Total Environ.

[CR66] Hunova I, Maznová J, Kurfurst P (2014). Trends in atmospheric deposition fluxes of sulphur and nitrogen in Czech forests. Environ Pollut.

[CR67] Hunova I (2020) Ambient air quality in the Czech Republic: past and present. Atmosphere 11(2):214. https://www.mdpi.com/2073-4433/11/2/214

[CR68] Jacobson AD, Holmden C (2008). δ^44^Ca evolution in a carbonate aquifer and its bearing on the equilibrium isotope fractionation factor for calcite. Earth Planet Sci Lett.

[CR69] Jacobson AD, Andrews MG, Lehn GO, Holmden C (2015). Silicate versus carbonate weathering in Iceland: new insights from Ca isotopes. Earth Planet Sc Lett.

[CR70] Janousek V, Erban Kochergina YV, Andronikov A, Kusbach V (2022). Decoupling of Mg from Sr–Nd isotopic compositions in Variscan subduction-related plutonic rocks from the Bohemian Massif: Implications for mantle enrichment processes and genesis of orogenic ultrapotassic magmatic rocks. Int J Earth Sci.

[CR71] Jochum KP, Nohl U (2008). Reference materials in geochemistry and environmental research and the GeoReM database. Chem Geol.

[CR72] Kalinowski BE, Schweda P (1996). Kinetics of muscovite, phlogopite, and biotite dissolution and alteration at pH 1–4, room temperature. Geochim Cosmochim Acta.

[CR73] Kimmig SR, Holmden C, Belanger N (2018). Biogeochemical cycling of Mg and its isotopes in a sugar maple forest in Quebec. Geochim Cosmochim Acta.

[CR74] Knauss KG, Wolery TJ (1986). Dependence of albite dissolution kinetics on pH and time at 25°C and 70°C. Geochim Cosmochim Acta.

[CR75] Kohler SJ, Dufaud F, Oelkers EH (2003). An experimental study of illite dissolution kinetics as a function of pH from 1.4 to 12.4 and temperatures from 5 to 50°C. Geochim Cosmochim Acta.

[CR76] Kohler L, Leuschner C, Hauck M, Hertel D (2015). Cloud water interception and element deposition differ largely between Norway spruce stands along an elevation transect in Harz Mountains, Germany. Ecohydrology.

[CR77] Kopacek J, Posch M, Hejzlar J, Oulehle F, Volkova A (2012). An elevation-based regional model for interpolating sulphur and nitrogen deposition. Atmos Environ.

[CR78] Kopacek J, Hejzlar J, Kram P, Oulehle F, Posch M (2016). Effect of industrial dust on precipitation chemistry in the Czech Republic (Central Europe) from 1850 to 2013. Water Res.

[CR79] Kram P, Hruška J, Shanley JB (2012). Streamwater chemistry in three contrasting monolithologic Czech catchments. Appl Geochem.

[CR80] Li D, Shields G, Ling HF, Thirlwall M (2010). Sequential leaching methods for strontium isotope stratigraphy: Analysis of marine authigenic carbonates and phosphates. Geochim Cosmochim Acta.

[CR81] Li MYH, Teng FZ, Zhou MF (2021). Phyllosilicate controls on magnesium isotopic fractionation during weathering of granites: Implications for continental weathering and riverine system. Earth Planet Sci Lett.

[CR82] Liu SA, Li SG (2019). Tracing the deep carbon cycle using metal stable isotopes: Opportunities and challenges. Engineering.

[CR83] Lucas Y, Chabaux F, Schaffhauser T, Fritz B, Ambroise B, Ackerer J, Clément A (2017). Hydrogeochemical modeling (KIRMAT) of spring and deep borehole water compositions in the small granitic Ringelbach catchment (Vosges Mountains, France). Appl Geochem.

[CR84] Ma L, Teng FZ, Jin L, Ke S, Yang W, Gu HO, Brantley SL (2015). Magnesium isotope fractionation during shale weathering in the Shale Hills Critical Zone Observatory: accumulation of light Mg isotopes in soils by clay mineral transformation. Chem Geol.

[CR85] Maguire ME, Cowan JA (2002). Magnesium chemistry and biochemistry. BioMetals.

[CR86] Maher K, von Blanckenburg F (2023). The circular nutrient economy of terrestrial ecosystems and the consequences for rock weathering. Front Environ Sci.

[CR87] Marschner H (1995) Mineral nutrition in higher plants. Academic Press San Diego, Elsevier Ltd., USA. 10.1016/B978-0-12-473542-2.X5000-7

[CR88] McArthur JM, Howarth RJ, Shields GA, Zhou Y (2020) Sr-isotope stratigraphy. Chapter 7, Vol. 1, 211–238 In: Gradstein FM, Ogg JG, Schmitz MD, Ogg G M (Eds), A Geologic Time Scale 2020. Vol 2, Elsevier, pp 1357. 10.1016/B978-0-12-824360-2.00007-3

[CR89] Mencik E and Pesl V (1995) Geological map on the scale 1:25 000, no. 2524 Turzovka and M-34–85-D-d (Bila). Czech Geological Survey

[CR90] Mencik E, Adamova M, Dvorak J, Dudek A, Jetel J, Jurkova A, Hanzlikova E, Housa V, Peslova H, Rybarova L, Smid B, Sebesta J, Tyracek J, Vasicek Z (1983) Geology of the Moravo-Silesian Beskydy Mts. Academia, pp 304

[CR91] Michel RL, Campbell D, Clow D, Turk JT (2000). Timescales for migration of atmospherically derived sulphate through an alpine/subalpine watershed, Loch Vale Colorado. Water Resour Res.

[CR92] Miller EK, Friedland AJ, Arons EA, Mohnen VA, Battles J, Panek JA, Kadlecek J, Johnson AH (1993). Atmospheric deposition to forests along an elevational gradient at Whiteface Mountain, NY, USA. Atmos Environ Part A Gen Top.

[CR93] Moldan B, Cerny J (1994) Biogeochemistry of small catchments – a tool for environmental research. Scope 51, John Wiley & Sons., Chichester, pp 419

[CR94] Moore J, Jacobson AD, Holmden C, Craw D (2013). Tracking the relationship between mountain uplift, silicate weathering, and long-term CO_2_ consumption with Ca isotopes: Southern Alps, New Zealand. Chem Geol.

[CR95] Nadaskay R, Kochergina YV, Cech S, Svabenicka L, Valecka J, Erban V, Halodova P, Cejkova B (2019). Integrated stratigraphy of an offshore succession influenced by intense siliciclastic supply: implications for Coniacian tectono-sedimentary evolution of the West Sudetic area (NW Bohemian Cretaceous Basin, Czech Republic). Cretaceous Res.

[CR96] Nezat CA, Blum JD, Yanai RD, Hamburg SP (2007). A sequential extraction to determine the distribution of apatite in granitoid soil mineral pools with application to weathering at the Hubbard Brook Experimental Forest, NH, USA. Appl Geochem.

[CR97] Nguyen TH, Watmough SA, Dang DH (2023). Evaluating the use of Ca/Sr and ^87^Sr/^86^Sr ratios to track Ca sources in sugar maple in Ontario. Can J Forest Res.

[CR98] Novak M, Andronikov A , Kram P, Curik J, Veselovsky F, Stepanova M, Prechova E, Sebek O and Bohdalkova L (2021) Time-series of δ26Mg values in a headwater catchment reveal decreasing magnesium isotope variability from recipitation to runoff Hydrol. Proces 35:e14116. 10.1002/hyp.14116

[CR99] Novak M, Bottrell SH, Fottova D, Buzek F, Groscheova H, Zak K (1996). Sulfur isotope signals in forest soils of Central Europe along an air-pollution gradient. Environ Sci Technol.

[CR100] Novak M, Kirchner J, Groscheova H, Cerny J, Havel M, Krejci R, Buzek F (2000). Sulfur isotope dynamics in two Central European watersheds affected by high atmospheric deposition of SO_x_. Geochim Cosmochim Acta.

[CR101] Novak M, Michel RL, Prechova E, Stepanova M (2004). The missing flux in a ^35^S budget for the soils of a small polluted catchment. Water Air Soil Pollut: Focus.

[CR102] Novak M, Kirchner JW, Fottova D, Prechova E, Jackova I, Kram P, Hruška J (2005). Isotopic evidence for processes of sulfur retention/release in 13 Central European catchments spanning a strong pollution gradient. Glob Biogeochem Cy.

[CR103] Novak M, Holmden C, Farkas J, Kram P, Hruška J, Curik J, Veselovsky F, Stepanova M, Kochergina YV, Erban V, Fottova D, Simecek M, Bohdalkova L, Prechova E, Voldrichova P, Cernohous V (2020a) Calcium and strontium isotope dynamics in three polluted forest ecosystems of the Czech Republic, Central Europe. Chem Geol 536(UNSP):119472. 10.1016/j.chemgeo.2020.119472

[CR104] Novak M, Farkas J, Kram P, Hruška J, Stepanova M, Veselovsky F, Curik J, Andronikov AV, Sebek O, Simecek M, Fottova D, Bohdalkova L, Prechova E, Koubova M, Vitkova H (2020b) Controls on δ^26^Mg variability in three Central European headwater catchments characterized by contrasting bedrock chemistry and contrasting inputs of atmospheric pollutants. Plos One 15(11)11):e0242915. 10.1371/journal.pone.0242915 10.1371/journal.pone.0242915 PMC7703950 33253305

[CR105] Novak M, Andronikov AV, Holmden Ch, Erban Kochergina YV, Veselovsky F, Paces T, Vitková M, Kachlik V, Sebek O, Hruška J, Stepanova M, Curik J, Prechova E, Fottova D, Andronikova IE, Erban V, Koubova M, Vostra I, Houskova M, Komarek A (2023a) δ^26^Mg, δ^44^Ca and ^87^Sr/^86^Sr isotope differences among bedrock minerals constrain runoff generation in headwater catchments: an acidified granitic site in Central Europe as an example. Catena 221:106780. 10.1016/j.catena.2022.106780

[CR106] Novak M, Holmden CH, Farkas J, Kram P, Hruška J, Curik J, Veselovsky F, Stepanova M, Kochergina YV, Erban V, Andronikov A, Sebek O, Koubova M, Bohdalkova L, Vitkova H (2020c) Magnesium and calcium isotope systematics in a headwater catchment underlain by amphibolite: Constraints on Mg–Ca biogeochemistry in an atmospherically polluted but well-buffered spruce ecosystem (Czech Republic, Central Europe). Catena 193. 10.1016/j.catena.2020.104637

[CR107] Novak M, Holmden CH, Andronikov AV, Erban Kochergina YV, Paces T, Kachlik V, Veselovsky F, Hruška J, Stepanova M, Prechova E, Sebek O, Curik J, Tesar M, Fottova D, Andronikova IE, Koubova M, Komarek A (2023b) Magnesium, calcium and strontium isotope dynamics in a small forested catchment underlain by paragneiss: the role of geogenic, atmospheric, and biogenic sources of base cations Geoderma 442:116768. 10.1016/j.geoderma.2023.116768

[CR108] Nuruzzama M, Rahaman W, Tripathy GR, Mohan R, Patil S (2020) Dissolved major ions, Sr and ^87^Sr/^86^Sr of coastal lakes from Larsemann Hills, East Antarctica: solute sources and chemical weathering in a polar environment. Hydrol Process 34(11):2351–2364. 10.1002/hyp.13734

[CR109] Oehlerich M, Mayr C, Gussone N, Hahn A, Hölzl S, Lücke A, Ohlendorf C, Rummel S, Teichert BMA, Zolitschka B (2015). Lateglacial and Holocene climatic changes in south-eastern Patagonia inferred from carbonate isotope records of Laguna Potrok Aike (Argentina). Quaternary Sci Rev.

[CR110] Oelkers EH, Schott J (1995). Experimental study of anorthite dissolution rates and the relative mechanism of feldspar hydrolysis. Geochim Cosmochim Acta.

[CR111] Opfergelt S, Georg RB, Delvaux B, Cabidoche Y-M, Burton KW, Halliday AN (2012). Mechanisms of magnesium isotope fractionation in volcanic soil weathering sequences, Guadeloupe. Earth Planet Sci Lett.

[CR112] Opfergelt S, Burton KW, Georg RB, West AJ, Guicharnaud RA, Sigfusson B, Siebert C, Gislason SR, Halliday AN (2014). Magnesium retention on the soil exchange complex controlling Mg isotope variations in soils, soil solutions and vegetation in volcanic soils, Iceland. Geochim Cosmochim Acta.

[CR113] Oulehle F, Hofmeister J, Cudlin P, Hruška J (2006). The effect of reduced atmospheric deposition on soil and soil solution chemistry at a site subjected to long-term acidification, Nacetin, Czech Republic. Sci Total Environ.

[CR114] Oulehle F, Hleb R, Houska J, Samonil P, Hofmeister J, Hruška J (2010). Anthropogenic acidification effects in primeval forests in the Transcarpathian Mts., western Ukraine. Sci Total Environ.

[CR115] Oulehle F, Chuman T, Hruška J, Kram P, McDowell WH, Myska O, Navratil T, Tesar M (2017). Recovery from acidification alters concentrations and fluxes of solutes from Czech catchments. Biogeochemistry.

[CR116] Oulehle F, Fischer M, Hruška J, Chuman T, Kram P, Navratil T, Tesar M, Trnka M (2021). The GEOMON network of Czech catchments provides long-term insights into altered forest biogeochemistry: from acid atmospheric deposition to climate change. Hydrol Process.

[CR117] Oursin M, Pierret MC, Beaulieu E, Daval D, Legout A (2023). Is there still something to eat for trees in the soils of the Strengbach catchment?. Forest Ecol Manag.

[CR118] Page BD, Bullen TD, Mitchell MJ (2008). Influences of calcium availability and tree species on Ca isotope fractionation in soil and vegetation. Biogeochemistry.

[CR119] Pin C, Gannoun A, Dupont A (2014). Rapid, simultaneous separation of Sr, Pb, and Nd by extraction chromatography prior to isotope ratios determination by TIMS and MC-ICP-MS. J Anal Atom Spectrom.

[CR120] Pogge von Strandmann PAE, Burton KW, James RH, van Calsteren P, Gislason SR, Sigfusson B (2008). The influence of weathering processes on riverine magnesium isotopes in a basaltic terrain. Earth Planet Sci Lett.

[CR121] Pogge von Strandmann PAE, Elliott T, Marschall HR, Coath C, Lai Y-J, Jeffcoate AB, Ionov DA (2011). Variations of Li and Mg isotope ratios in bulk chondrites and mantle xenoliths. Geochim Cosmochim Acta.

[CR122] Pokharel R, Gerrits R, Schuessler JA, Floor GH, Gorbushina AA, von Blankenburg F (2017). Mg isotope fractionation during uptake by a rock-inhabiting, model microcolonial fungus *Knufia petricola* at acidic and neutral pH. Environ Sci Technol.

[CR123] Prechova E, Sebek O, Strnad L, Novak M, Chrastny V, Stepanova M, Pasava J, Veselovsky F, Curik J, Pacherova P, Bohdalkova L, Houskova M (2020). Temporal changes in mountain-slope gradients in the concentrations of pollutants and Pb isotope ratios near the Ostrava conurbation (Upper Silesia, Czech-Polish Border). Water Air Soil Pollut..

[CR124] Prechova E, Sebek O, Novak M, Andronikov AV, Strnad L, Chrastny V, Cabala J, Stepanova M, Pasava J, Martinkova E, Pacherova P, Blaha V, Curik J, Veselovsky F, Vitkova H (2023). Spatial and temporal trends in δ^66^Zn and ^206^Pb/^207^Pb isotope ratios along a rural transect downwind from the Upper Silesian industrial area: role of legacy vs. present-day pollution. Environ Pollut.

[CR125] Probst A, Dambrine E, Viville D, Fritz B (1990). Influence of acid atmospheric inputs on surface water chemistry and mineral fluxes in a declining spruce stand within a small granitic catchment (Vosges Massif, France). J Hydrol.

[CR126] Pu G, Campbell JL, Green MB, Merriam JL, Zietlow D, Yanai RD (2023). Estimating uncertainty in streamflow and solute fluxes at the Hubbard Brook Experimental Forest, New Hampshire, USA. Hydrol Process.

[CR127] Riebe CS, Kirchner JW, Finkel RC (2004). Erosional and climatic effects on long-term chemical weathering rates in granitic landscapes spanning diverse climate regimes. Earth Planet Sci Lett.

[CR128] Ryan SE, Snoeck C, Crowley QG, Babechuk MG (2018). ^87^Sr/^86^Sr and trace element mapping of geosphere–hydrosphere–biosphere interactions: a case study in Ireland. Appl Geochem.

[CR129] Ryu J-S, Jacobson AD, Holmden C, Lundstrom C, Zhang Z (2011). The major ion, δ^44/40^Ca, δ^44/42^Ca, and δ^26/24^Mg geochemistry of granite weathering at pH = 1 and T = 25 °C: power-law processes and the relative reactivity of minerals. Geochim Cosmochim Acta.

[CR130] Ryu JS, Vigier N, Derry L, Chadwick OA (2021). Variations of Mg isotope geochemistry in soils over a Hawaiian 4 Myr chronosequence. Geochim Cosmochim Acta.

[CR131] Schmitt A-D, Gussone N, Schmitt A-D, Heuser A, Wombacher F, Dietzel M, Tipper E, Schiller M (2016). Earth-surface Ca isotopic fractionations. Calcium Stable Isotope Geochemistry.

[CR132] Schuessler JA, von Blanckenburg F, Bouchez J, Uhlig D, Hewawasam T (2018). Nutrient cycling in a tropical montane rainforest under a supply-limited weathering regime traced by elemental mass balances and Mg stable isotopes. Chem Geol.

[CR133] Schwartz JS, Veeneman A, Kulp MA, Renfro JR (2022). Throughfall deposition chemistry in the Great Smoky Mountains National Park: Landscape and Seasonal Effects. Water Air Soil Pollut.

[CR134] Shalev N, Farkas J, Fietzke J, Novak M, Schuessler JA, Pogge von Strandmann PAE, Torber PB (2018). Mg isotope interlaboratory comparison of reference materials from earth-surface low-temperature environments. Geostand Geoanal Res.

[CR135] Siegel DI, Pfannkuch HO (1984). Silicate mineral dissolution at pH 4 and near standard temperature and pressure. Geochim Cosmochim Acta.

[CR136] Soukhovolsky V, Kovalev A, Tarasova O, Modlinger R, Krenova Z, Mezei P, Skvarenina J, Roznovsky J, Korolyova N, Majdak A, Jakus R (2022). Wind damage and temperature effect on tree mortality caused by *Ips*
*typographus*
*L*: Phase transition model. Forests.

[CR137] Sterner R (2002) Ecological stoichiometry: the biology of elements from molecules to the biosphere. In: Ecological stoichiometry, Princeton, Princeton University Press. 10.1515/9781400885695

[CR138] Stranik Z, Bubik M, Gilikova H, Tomanova Petrova P (Eds) (2021) Geology of the Outer Western Carpathians, Czech Republic. Czech Geological Survey, p 320. ISBN 978–80–7075–061–2

[CR139] Sueker JK, Turk JT, Michel RL (1999). Use of cosmogenic ^35^S for comparing ages of water from three alpine–subalpine basins in the Colorado Front Range. Geomorphology.

[CR140] Swoboda-Colberg NG, Drever JI (1993). Mineral dissolution rates in plot-scale field and laboratory experiments. Chem Geol.

[CR141] Tang J, Dietzel M, Böhm F, Köhler SJ, Eisenhauer A (2008). Sr^2+^/Ca^2+^ and ^44^Ca/^40^Ca fractionation during inorganic calcite formation: II. Ca isotopes. Geochim Cosmochim Acta.

[CR142] Teng F-Z, Li W-Y, Ke S, Yang W, Liu S-A, Sedaghatpour F, Wang S-J, Huang K-J, Hu Y, Ling M-X, Xiao Y, Liu X-M, Li X-W, Gu H-O, Sio CK, Wallace DA, Su B-X, Zhao L, Chamberlin J, Harrington M, Brewer A (2015) Magnesium isotopic compositions of international geological reference materials. Geostand Geoanal Res 39:329–339. 10.1111/j.1751-908X.2014.00326.x

[CR143] Teng F-Z, Watkins JM, Dauphas N (2017) Non-traditional Stable Isotopes, Reviews in Mineralogy and Geochemistry, vol. 82, Mineralogical Society of America, Geochemical Society, p 885

[CR144] Teng F-Z (2017) Magnesium isotope geochemistry In: Teng F-Z, Watkins JM, Dauphas N (Eds), Non-traditional stable isotopes, reviews in Mineralogy and Geochemistry, vol. 82, Mineralogical Society of America, Geochemical Society, pp 219–287. 10.2138/rmg.2017.82.7

[CR145] Tipper ET, Galy A, Bickle MJ (2006). Riverine evidence for a fractionated reservoir of Ca and Mg on the continents: implications for the oceanic Ca cycle. Earth Planet Sci Lett.

[CR146] Tipper ET, Galy A, Bickle MJ (2008). Calcium and magnesium isotope systematics in rivers draining the Himalaya-Tibetan-Plateau region: lithological or fractionation control?. Geochim Cosmochim Acta.

[CR147] Tipper ET, Calmels D, Gaillardet J, Louvat P, Capmas F, Dubacq B (2012). Positive correlation between Li and Mg isotope ratios in the river waters of the Mackenzie Basin challenges the interpretation of apparent isotopic fractionation during weathering. Earth Planet Sci Lett.

[CR148] Tipper E, Schmitt AD, Gussone N, Gussone N, Schmitt A-D, Heuser A, Wombacher F, Dietzel M, Tipper E, Schiller M (2016). Global Ca cycles: Coupling of continental and oceanic processes. Calcium Stable Isotope Geochemistry.

[CR149] Tukey JW (1953) The problem of multiple comparisons In: Braun HI (Ed), The Collected Works of John W. Tukey, volume 8, 1994. Chapman & Hall, New York. ISBN 9780412051210

[CR150] van der Heijden G, Legout A, Pollier B, Ranger J, Dambrine E (2014). The dynamics of calcium and magnesium inputs by throughfall in a forest ecosystem on base poor soil are very slow and conservative: evidence from an isotopic tracing experiment (^26^Mg and ^44^Ca). Biogeochemistry.

[CR151] van der Heijden G, Dambrine E, Pollier B, Zeller B, Ranger J, Legout A (2015). Mg and Ca uptake by roots in relation to depth and allocation to aboveground tissues: results from an isotopic labeling study in a beech forest on base-poor soil. Biogeochemistry.

[CR152] Vicha Z (2019). Evaluation of the hydrological year 2017 in experimental basins Cervik and Mala Raztoka, the Moravian-Silesian Beskids, Czech Republic. Rep For Res.

[CR153] Voldrichova P, Chrastny V, Sipkova A, Farkas J, Novak M, Stepanova M, Krachler M, Veselovsky F, Blaha V, Prechova E, Komarek A, Bohdalkova L, Curik J, Mikova J, Erbanova L, Pacherova P (2014). Zinc isotope systematics in snow and ice accretions in Central European mountains. Chem Geol.

[CR154] von Blanckenburg F, Schuessler JA, Bouchez J, Frings PJ, Uhlig D, Oelze M, Frick DA, Hewawasam T, Dixon J, Norton K (2021). Rock weathering and nutrient cycling along an erodosequence. Am J Sci.

[CR155] Welch SA, Ullman WJ (1996). Feldspar dissolution in acidic and organic solutions. Compositional and pH dependence of dissolution rate. Geochim Cosmochim Acta.

[CR156] Wiegand BA, Chadwick OA, Vitousek PM, Wooden JL (2005) Ca cycling and isotopic fluxes in forested ecosystems in Hawaii. Geophys Res Lett 32(11). 10.1029/2005GL022746.

[CR157] Wiggenhauser M, Moore RE, Wang P, Bienert GP, Laursen KH, Blotevogel S (2022). Stable isotope fractionation of metals and metalloids in plants: a review. Front Plant Sci.

[CR158] Wimpenny J, Burton KW, James RH, Gannoun A, Mokadem F, Gíslason SR (2011). The behaviour of magnesium and its isotopes during glacial weathering in an ancient shield terrain in West Greenland. Earth Planet Sci Lett.

[CR159] Wolff-Boenisch D, Gislason SR, Oelkers EH (2006) The effect of crystallinity on dissolution rates and CO_2_ consumption capacity of silicates. Geochim Cosmochim Acta 70(4):858–870. 10.1016/j.gca.2005.10.016

[CR160] Xu Y, Jin Z, Gou LF, Galy A, Jin C, Chen C, Li Ch, Deng L (2022). Carbonate weathering dominates magnesium isotopes in large rivers: clues from the Yangtze River. Chem Geol.

[CR161] Zahradnik P, Zahradnikova M (2023) Dynamics of emergent clearings during the current bark-beetle calamity. In: Plans on reforestation of bark-beetle induced open areas, P Zahradnik and Z Vacek (eds.) National Museum of Agriculture, Czech Forestry Society, pp 7–14

[CR162] Zhao T, Liu W, Xu Z, Sun H, Zhou X, Zhou L, Zang J, Zhang X, Jiang H, Liu T (2019). The influence of carbonate precipitation on riverine magnesium isotope signals: new constrains from Jinsha River Basin, Southeast Tibetan Plateau. Geochim Cosmochim Acta.

[CR163] Zhao T, Liu W, Li Y, Xu Z (2022). Magnesium isotopic composition of rivers draining karst-dominated regions in Southwest China. Chem Geol.

[CR164] Zhao T, Liu W, Xu Z (2022). Magnesium isotope fractionation during silicate weathering: constrains from riverine Mg isotopic composition in the southeastern coastal region of China. Geochem Geophys Geosyst.

