# Supplementary figures and images for: Sizeable net export of base cations from a Carpathian flysch catchment indicates their geogenic origin while the 26Mg/24Mg, 44Ca/40Ca and 87Sr/86Sr isotope ratios in runoff are indistinguishable from atmospheric input
Source: Environ Sci Pollut Res Int. 2024 Mar 18;31(17):26261–81. doi: 10.1007/s11356-024-32866-1 (PMC11024055; doi:10.1007/s11356-024-32866-1)

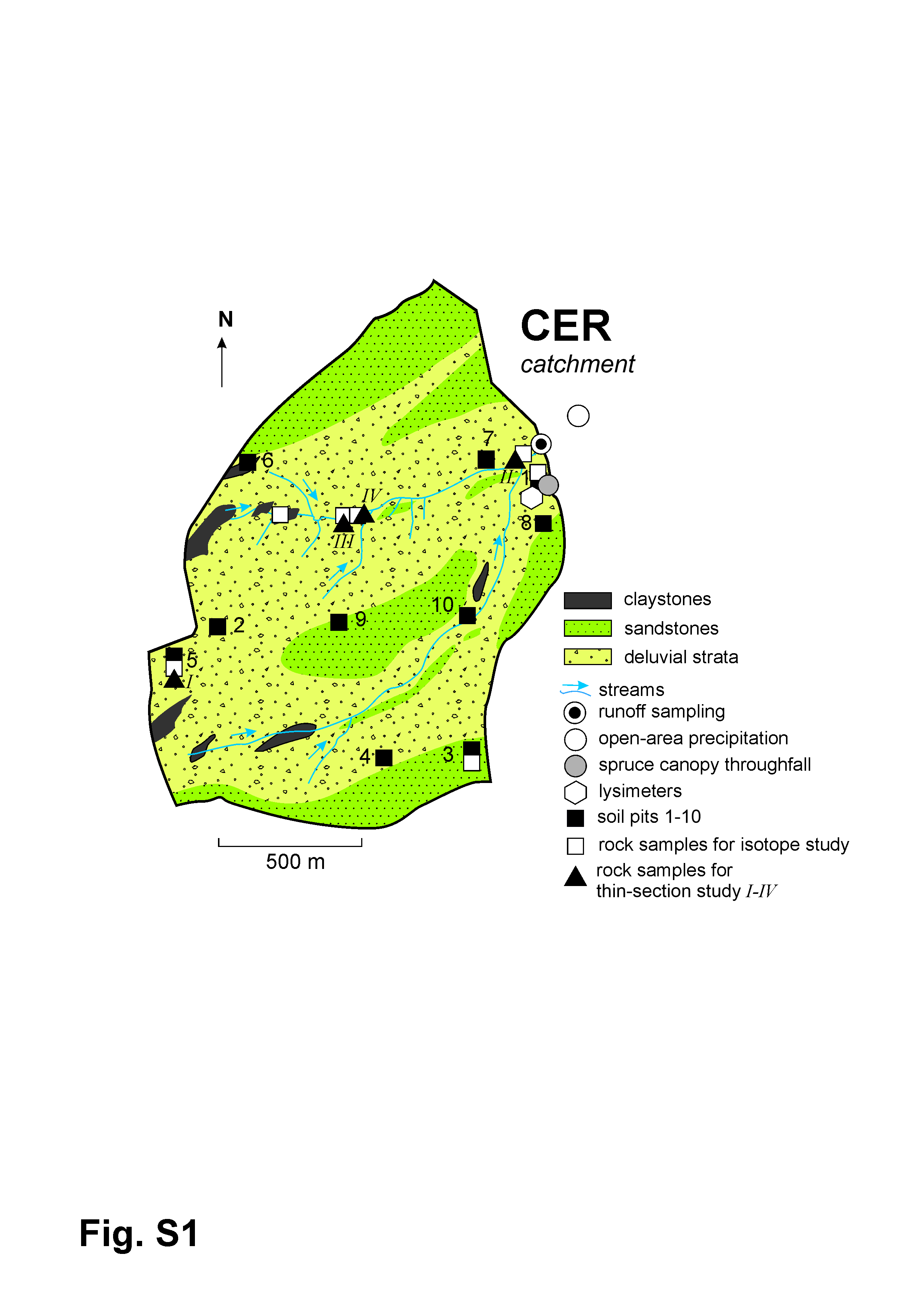

Supplement: Supplementary file 1 — Supplementary file1 Fig. S1. Geological sketch of the CER catchment (Mencik and Pesl 1995). (TIF 35872 KB) [file 11356_2024_32866_MOESM1_ESM.tif]

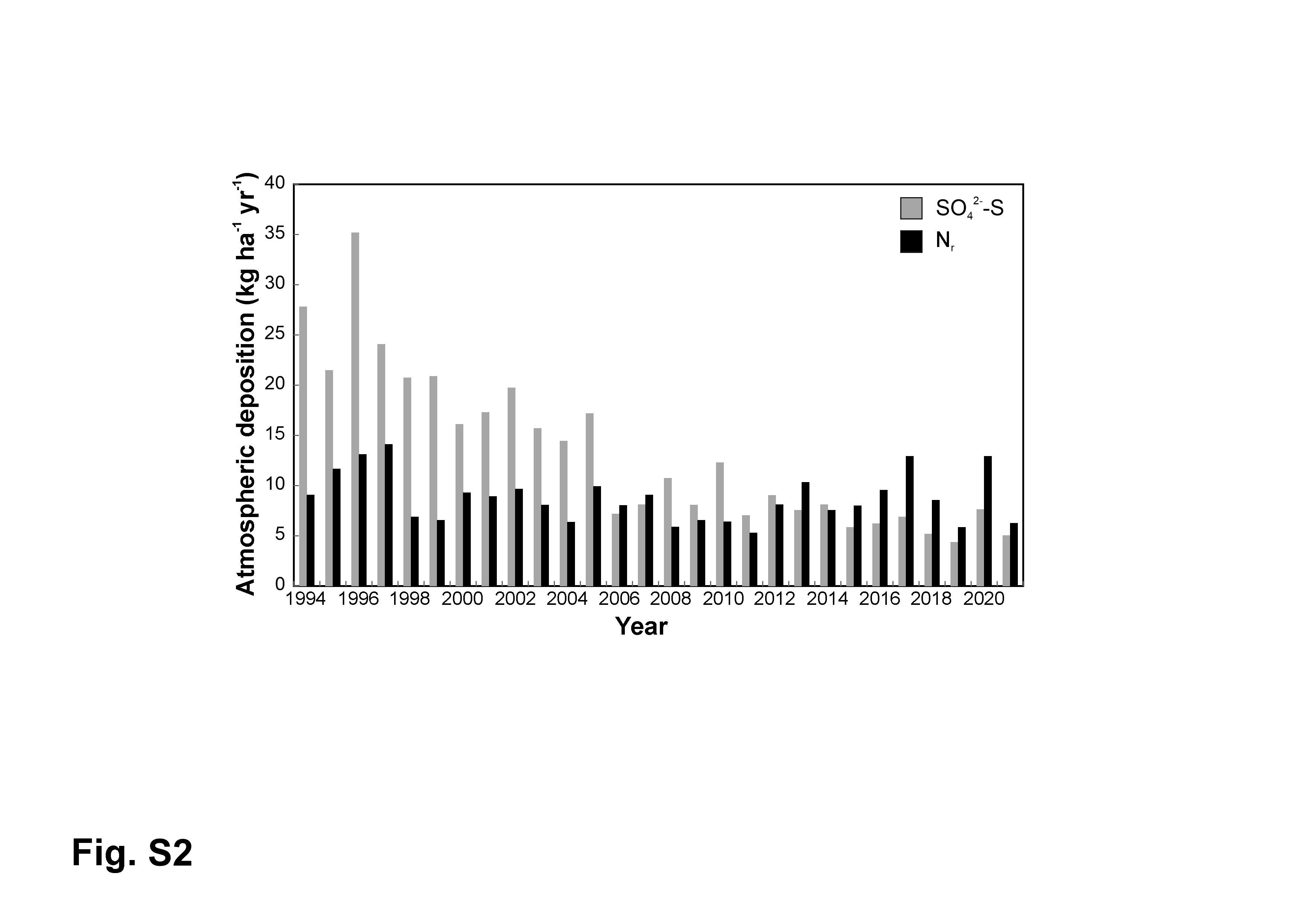

Supplement: Supplementary file 2 — Supplementary file2 Fig. S2. Annual S and Nr depositions at CER (Oulehle et al 2021). (TIF 8531 KB) [file 11356_2024_32866_MOESM2_ESM.tif]

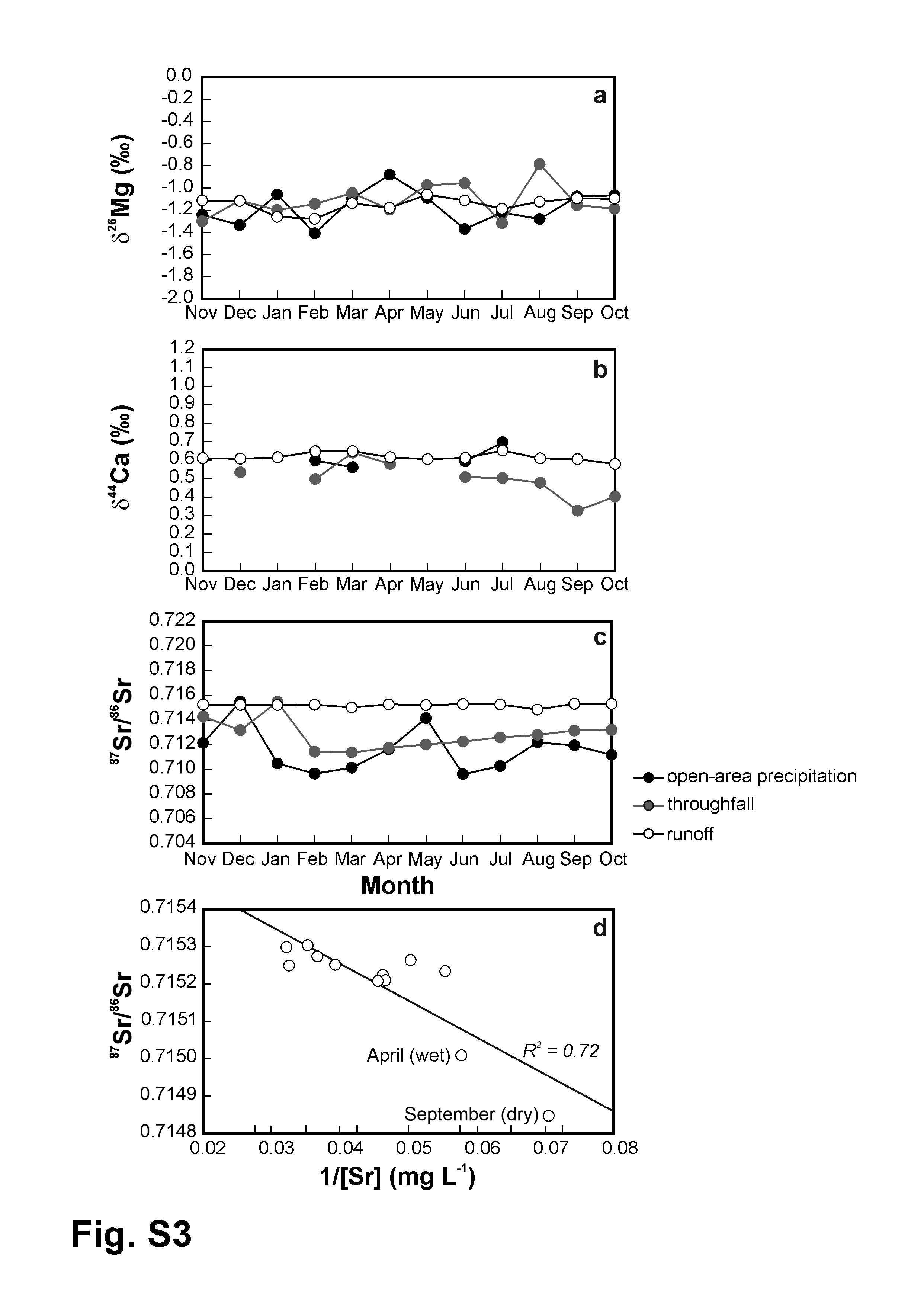

Supplement: Supplementary file 3 — Supplementary file3 Fig. S3. Monthly time-series of δ26Mg, δ44Ca and 87Sr/86Sr ratios in catchment inputs and output (a-c). Negative correlation between 1/[Sr] and 87Sr/86Sr in runoff (d). No such relationships were observed for Ca and Mg. (TIF 8528 KB) [file 11356_2024_32866_MOESM3_ESM.tif]

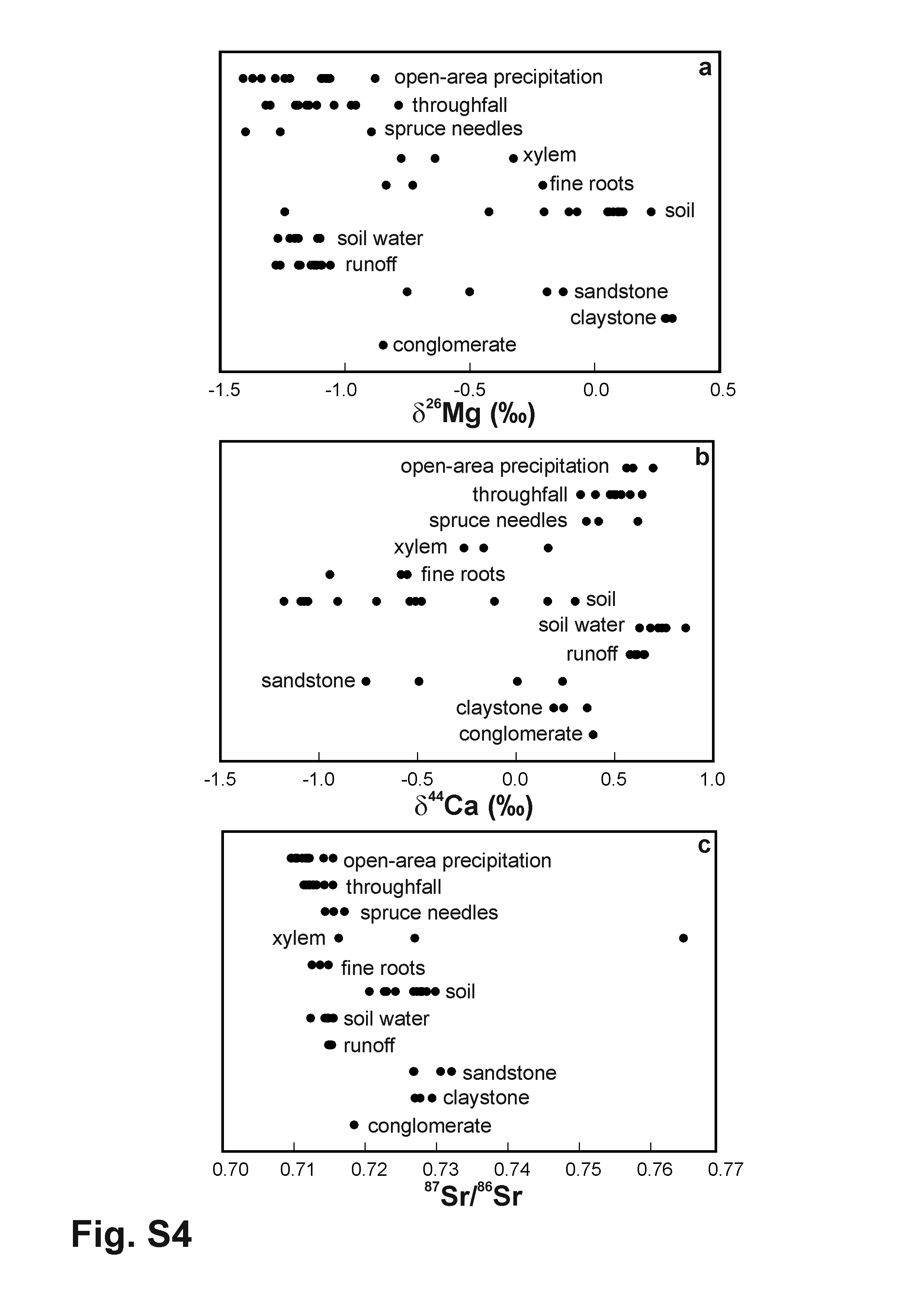

Supplement: Supplementary file 4 — Supplementary file4 Fig. S4. Individual isotope measurements used in Fig. 2. (TIF 8528 KB) [file 11356_2024_32866_MOESM4_ESM.tif]

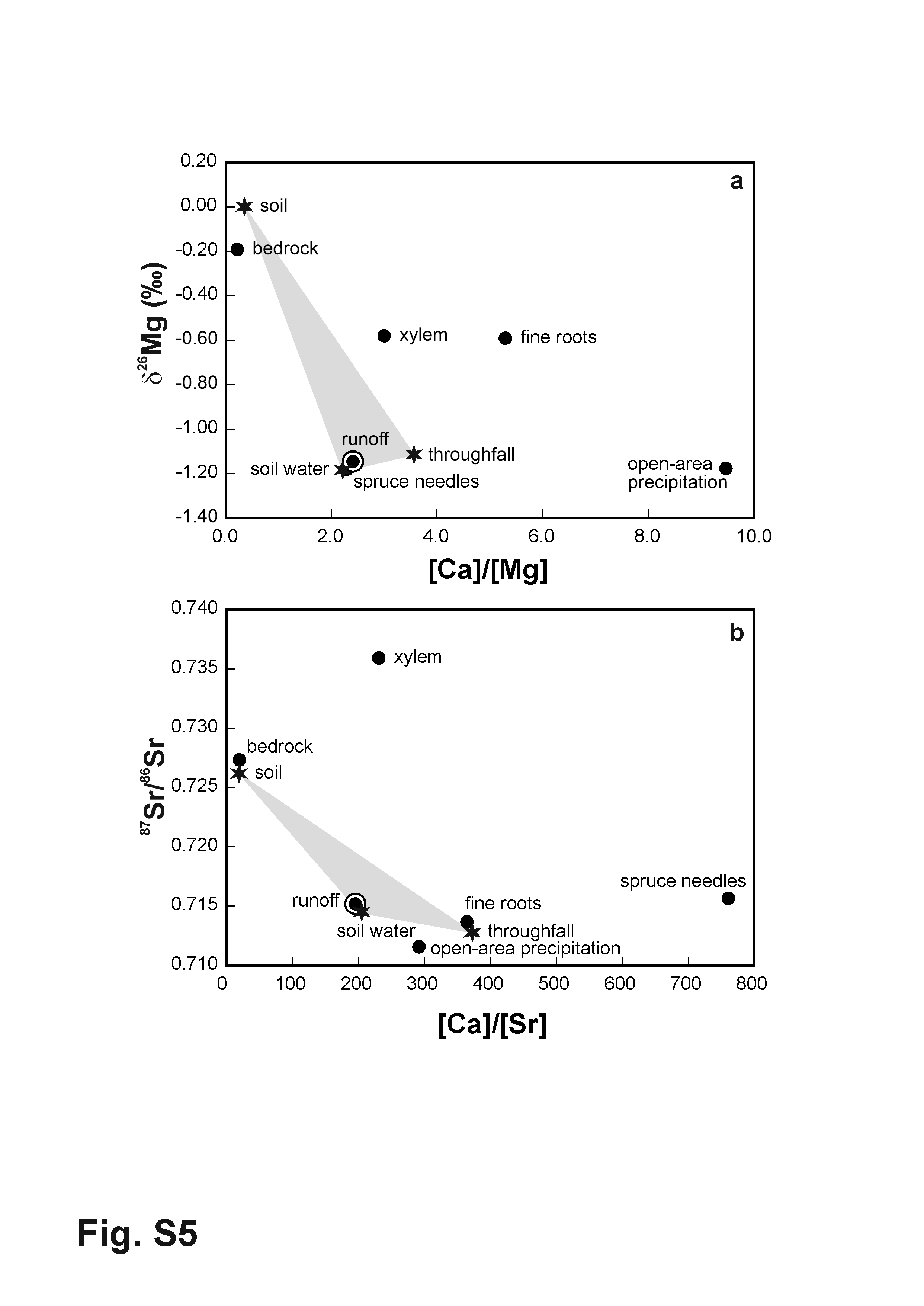

Supplement: Supplementary file 5 — Supplementary file5 Fig. S5. Mixing plots according to Capo et al (1998) using throughfall, soil water and bulk soil as the mixing endmembers. In both models, soil water appeared to contribute more than 90 % of the studied base cations to runoff. These models provide qualitative rather than quantitative estimates because of the known non-conservative behavior of some of the tracers and incompatible matrix in liquid vs. solid concentration measurements. For more detailed discussion of the limitations of the endmember mixing plots see, e.g., Novak et al (2020c). (TIF 8528 KB) [file 11356_2024_32866_MOESM5_ESM.tif]

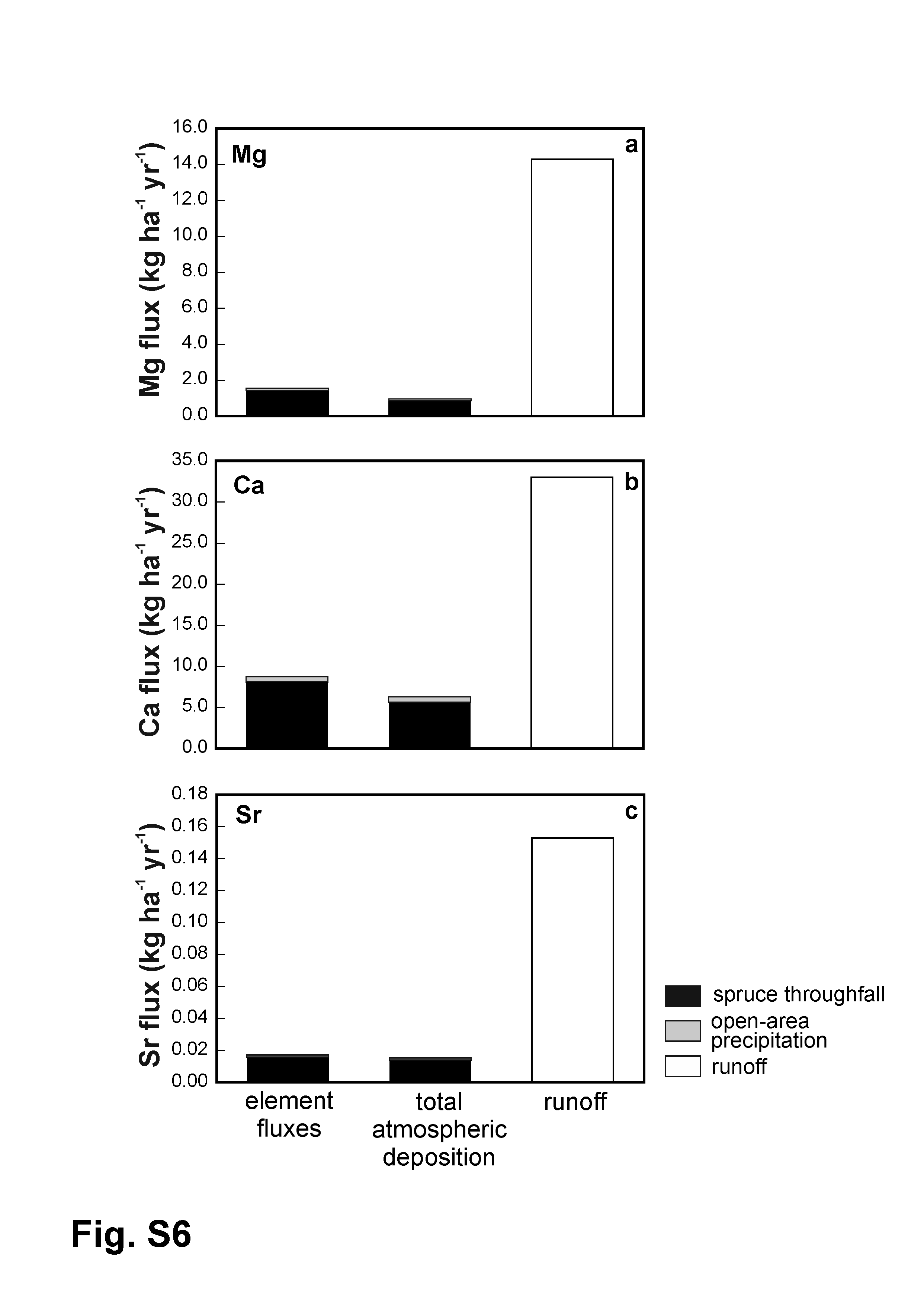

Supplement: Supplementary file 6 — Supplementary file6 Fig. S6. Comparison of average annual input and output fluxes of Mg, Ca, and Sr throughout the observation period. Conversion of the measured element fluxes (left bar) from Fig. 4 to total atmospheric deposition (middle bar) eliminated the effect of scavenging and leaching of base cations by canopy (Kopacek et al., 2016). (TIF 8528 KB) [file 11356_2024_32866_MOESM6_ESM.tif]

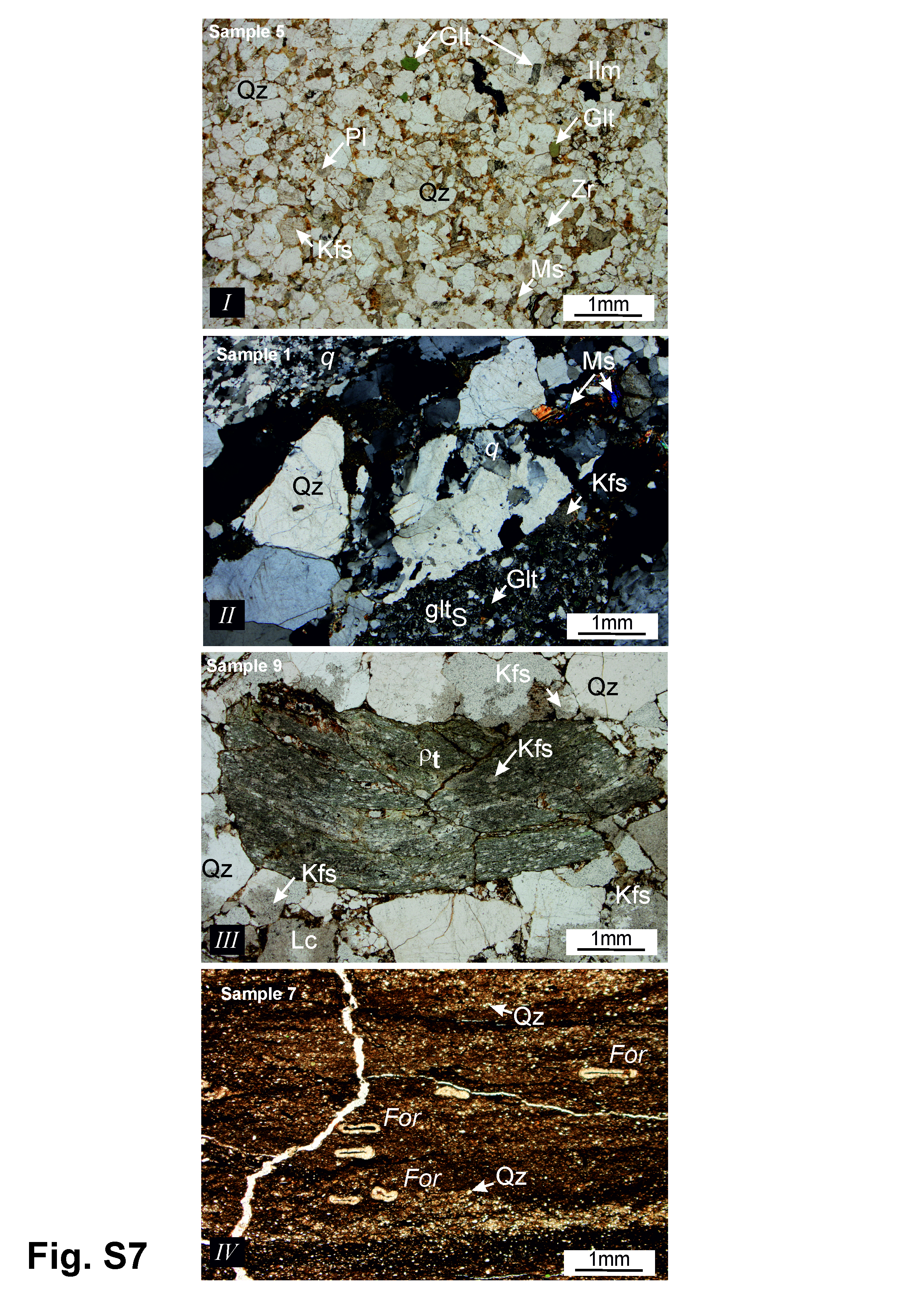

Supplement: Supplementary file 7 — Supplementary file7 Fig. S7. Mic rophotographs of thin sections from CER bedrock. I – coarse-grained arcosic sandstone with glauconite (parallel nicols); II – lithic conglomeratic sandstone to fine-grained conglomerate with clast of glauconitic sandstone, quartzite and mylonitized quartz grains and clastic white mica in the matrix (crossed nicols); III – coarse-grained lithic sandstone to fine-grained conglomeratic sandstone rich in lithic clast of magmatic and metamorphic rocks. Small pebble of rhyolite crystalloclastic tuff (parallel nicols); IV – laminated dark gray-green aleuropelite with quartz-dominated silty admixture and preserved silicified relics of benthic foraminifera of the genus Bathysiphon (parallel nicols). (TIF 35872 KB) [file 11356_2024_32866_MOESM7_ESM.tif]

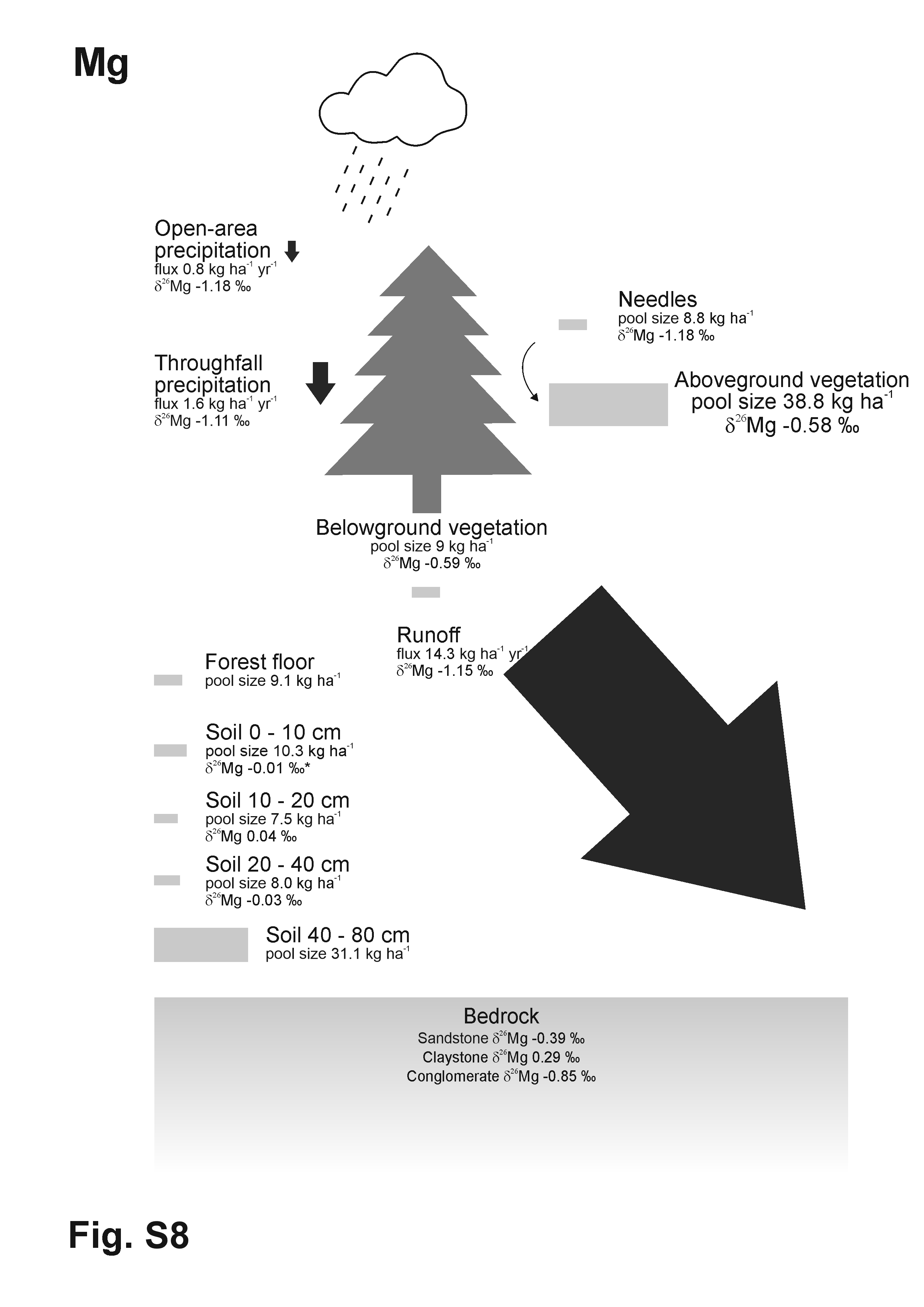

Supplement: Supplementary file 8 — Supplementary file8 Fig. S8. Mg pool size inventory in the CER catchment. (TIF 8530 KB) [file 11356_2024_32866_MOESM8_ESM.tif]

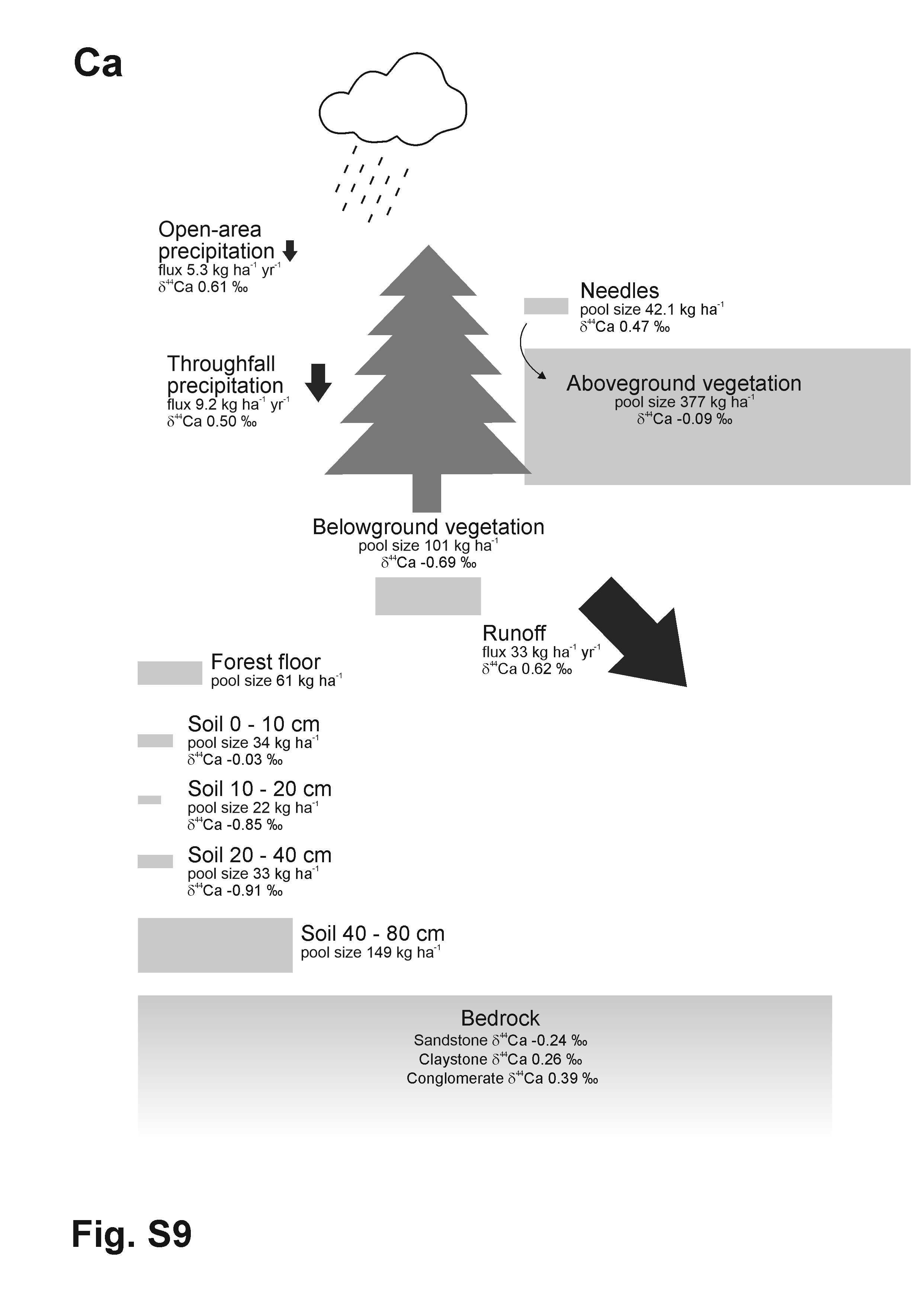

Supplement: Supplementary file 9 — Supplementary file9 Fig. S9. Ca pool size inventory in the CER catchment. (TIF 8528 KB) [file 11356_2024_32866_MOESM9_ESM.tif]

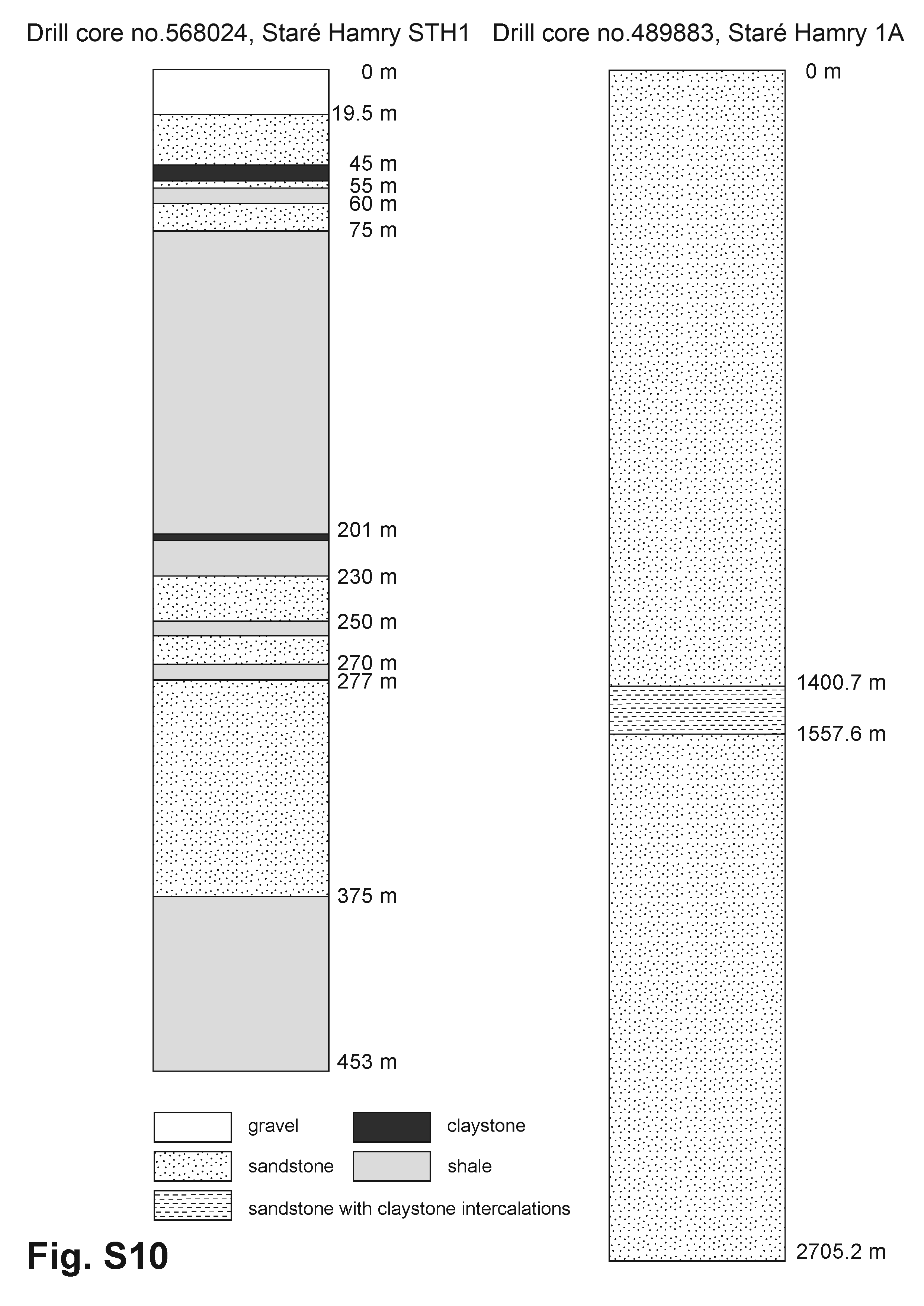

Supplement: Supplementary file 10 — Supplementary file10 Fig. S10. Schematic representation of the lithology of two drill cores from Stare Hamry, 4 km east of CER. ‘Geofond’ archive of the Czech Geological Survey, Prague. (TIF 8528 KB) [file 11356_2024_32866_MOESM10_ESM.tif]

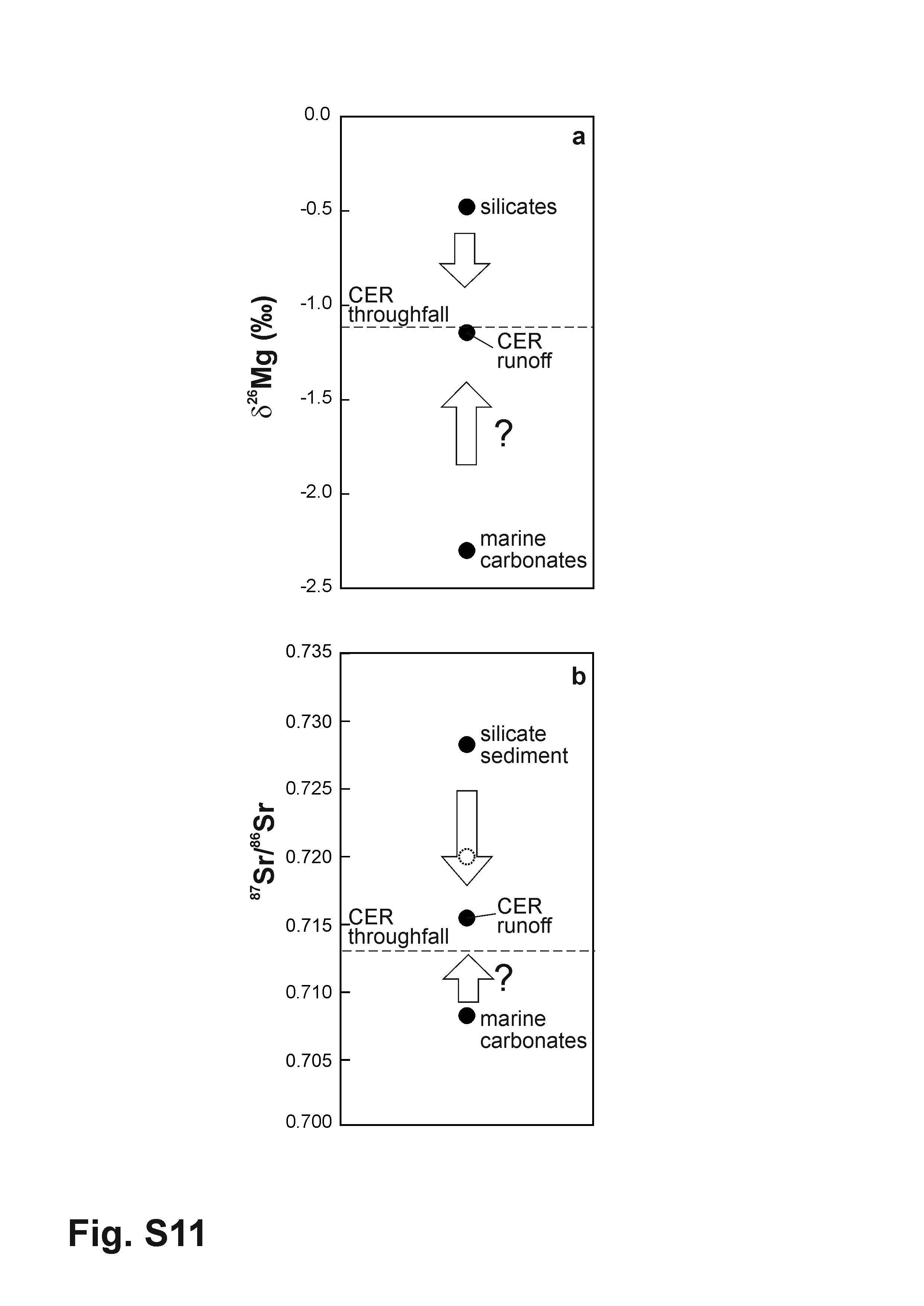

Supplement: Supplementary file 11 — Supplementary file11 Fig. S11. Hypothetical mixing of silicate and carbonate sources of Mg (a) and Sr (b) plotted together with CER runoff. The graphs use global mean δ26Mg and 87Sr/86Sr isotope ratios for each rock type, except for Sr in silicate sediments for which the global range of values is extremely broad, depending on age and Rb contents (0.705-0.750; Faure and Mensing 2004). Silicate sediment in panel (b) is represented by CER claystone (solid circle) and Central European marine sediments of a similar age (80-60 My BP; Nadaskay et al 2019; dotted circle). In the case of both elements, CER runoff plots between the higher isotope ratios of silicates and lower isotope ratios of carbonates, and, in principle, can be derived from dissolution of these two geogenic sources. Mean isotope signatures of mixing endmembers according to Gussone et al (2016), Teng (2017) and Zhao (2022a) and references therein. Isotope ratios in CER throughfall are marked by a horizontal dashed line. (TIF 8528 KB) [file 11356_2024_32866_MOESM11_ESM.tif]
