# Supplementary material for: Sizeable net export of base cations from a Carpathian flysch catchment indicates their geogenic origin while the 26Mg/24Mg, 44Ca/40Ca and 87Sr/86Sr isotope ratios in runoff are indistinguishable from atmospheric input
Source: Environ Sci Pollut Res Int. 2024 Mar 18;31(17):26261–81. doi: 10.1007/s11356-024-32866-1 (PMC11024055; doi:10.1007/s11356-024-32866-1)
Supplement: Supplementary file 12 — Supplementary file12 (DOCX 24 KB) [file 11356_2024_32866_MOESM12_ESM.docx]

**Table S1**. Mg and Ca concentrations and isotope composition of various types of samples.

| **Sample type** | **Depth in soil (cm)** | **Sampling date** | **Mg concentration (mg L^-1^/mg kg^-1^)** | **δ^26^Mg relative to DSM (‰)** | **Ca concentration**  **(mg L^-1^/mg kg^-1^)** | **δ^44^Ca relative to NIST915a (‰)** | **δ^44^Ca relative to seawater (‰)** |
| --- | --- | --- | --- | --- | --- | --- | --- |
| Open-area precipitation |  | November 2020 | 0.03 mg L^-1^ | -1.24 ± 0.03 | 0.29 mg L^-1^ | n.d. | n.d. |
| Spruce canopy throughfall |  | November 2020 | 0.05 | -1.30 ± 0.03 | 0.27 | n.d. | n.d. |
| Runoff |  | November 2020 | 2.11 | -1.11 ± 0.04 | 5.69 | 0.61 ± 0.07 | -1.24 |
| Open-area precipitation |  | December 2020 | 0.06 | -1.34 ± 0.02 | 0.33 | n.d. | n.d. |
| Spruce canopy throughfall |  | December 2020 | 0.14 | -1.11 ± 0.05 | 0.40 | 0.53 ± 0.05 | -1.32 |
| Runoff |  | December 2020 | 1.76 | -1.12 ± 0.06 | 4.40 | 0.61 ± 0.05 | -1.24 |
| Open-area precipitation |  | January 2021 | 0.08 | -1.06 ± 0.03 | 0.31 | n.d. | n.d. |
| Spruce canopy throughfall |  | January 2021 | 0.05 | -1.20 ± 0.03 | 0.33 | n.d. | n.d. |
| Runoff |  | January 2021 | 1.73 | -1.26 ± 0.06 | 4.02 | 0.62 ± 0.03 | -1.24 |
| Open-area precipitation |  | February 2021 | 0.11 | -1.41 ± 0.02 | 0.73 | 0.60 ± 0.03 | -1.25 |
| Spruce canopy throughfall |  | February 2021 | 0.13 | -1.14 ± 0.03 | 0.70 | 0.50 ± 0.04 | -1.35 |
| Runoff |  | February 2021 | 1.51 | -1.28 ± 0.05 | 4.51 | 0.65 ± 0.04 | -1.20 |
| Open-area precipitation |  | March 2021 | 0.08 | -1.10 ± 0.03 | 0.64 | 0.56 ± 0.03 | -1.29 |
| Spruce canopy throughfall |  | March 2021 | 0.26 | -1.04 ± 0.05 | 0.82 | 0.64 ± 0.03 | -1.21 |
| Runoff |  | March 2021 | 1.32 | -1.14 ± 0.06 | 3.58 | 0.65 ± 0.03 | -1.20 |
| Open-area precipitation |  | April 2021 | 0.05 | -0.88 ± 0.03 | 0.31 | n.d. | n.d. |
| Spruce canopy throughfall |  | April 2021 | 0.19 | -1.19 ± 0.03 | 0.62 | 0.58 ± 0.06 | -1.27 |
| Runoff |  | April 2021 | 1.62 | -1.18 ± 0.05 | 3.95 | 0.62 ± 0.03 | -1.24 |
| Open-area precipitation |  | May 2021 | 0.05 | -1.09 ± 0.03 | 0.28 | n.d. | n.d. |
| Spruce canopy throughfall |  | May 2021 | 0.18 | -0.98 ± 0.05 | 0.61 | n.d. | n.d. |
| Runoff |  | May 2021 | 1.77 | -1.06 ± 0.05 | 4.14 | 0.61 ± 0.04 | -1.25 |
| Open-area precipitation |  | June 2021 | 0.08 | -1.37 ± 0.03 | 0.83 | 0.59 ± 0.03 | -1.26 |
| Spruce canopy throughfall |  | June 2021 | 0.27 | -0.96 ± 0.05 | 0.96 | 0.51 ± 0.03 | -1.34 |
| Runoff |  | June 2021 | 2.18 | -1.11 ± 0.05 | 4.84 | 0.61 ± 0.03 | -1.24 |
| Open-area precipitation |  | July 2021 | 0.08 | -1.22 ± 0.04 | 2.56 | 0.70 ± 0.04 | -1.15 |
| Spruce canopy throughfall |  | July 2021 | 0.37 | -1.32 ± 0.05 | 1.07 | 0.50 ± 0.03 | -1.35 |
| Runoff |  | July 2021 | 2.44 | -1.19 ± 0.05 | 5.83 | 0.65 ± 0.03 | -1.20 |
| Open-area precipitation |  | August 2021 | 0.01 | -1.28 ± 0.03 | 0.11 | n.d. | n.d. |
| Spruce canopy throughfall |  | August 2021 | 0.17 | -0.79 ± 0.03 | 0.54 | 0.48 ± 0.03 | -1.37 |
| Runoff |  | August 2021 | 0.95 | -1.12 ± 0.05 | 2.43 | 0.61 ± 0.05 | -1.24 |
| Open-area precipitation |  | September 2021 | 0.02 | -1.08 ± 0.03 | 0.17 | n.d. | n.d. |
| Spruce canopy throughfall |  | September 2021 | 0.11 | -1.15 ± 0.04 | 0.46 | 0.33 ± 0.04 | -1.52 |
| Runoff |  | September 2021 | 2.39 | -1.09 ± 0.03 | 5.08 | 0.61 ± 0.03 | -1.25 |
| Open-area precipitation |  | October 2021 | 0.06 | -1.07 ± 0.03 | 0.24 | n.d. | n.d. |
| Spruce canopy throughfall |  | October 2021 | 0.14 | -1.19 ± 0.03 | 0.58 | 0.40 ± 0.05 | -1.45 |
| Runoff |  | October 2021 | 2.62 | -1.10 ± 0.03 | 5.51 | 0.58 ± 0.03 | -1.27 |
| Soil water 1 | -60 | Jun-Aug 2021 | 0.43 | -1.11 ± 0.03 | 1.00 | 0.74 ± 0.07 | -1.11 |
| Soil water 2 | -60 | Jun-Aug 2021 | 0.54 | -1.10 ± 0.05 | 1.15 | 0.72 ± 0.03 | -1.13 |
| Soil water 3 | -60 | Jun-Aug 2021 | 0.48 | -1.22 ± 0.04 | 1.73 | 0.76 ± 0.03 | -1.09 |
| Soil water 1 | -60 | Sep-Nov 2021 | 0.44 | -1.19 ± 0.05 | 0.76 | 0.63 ± 0.03 | -1.22 |
|  |  |  |  |  |  |  |  |
| Soil water 2 | -60 | Sep-Nov 2021 | 0.53 | -1.27 ± 0.05 | 1.01 | 0.68 ± 0.05 | -1.17 |
| Soil water 3 | -60 | Sep-Nov 2021 | 0.52 | -1.20 ± 0.04 | 1.05 | 0.86 ± 0.06 | -0.99 |
| Mineral soil 1 | 0-10 | October 2014 | 721 mg kg^-1^ | 0.05 ± 0.03 | 1160 mg kg^-1^ | 0.16 ± 0.03 | -1.69 |
| Mineral soil 1 | 10-20 | October 2014 | 1570 | 0.10 ± 0.03 | 212 | -0.54 ± 0.06 | -2.39 |
| Mineral soil 1 | 20-40 | October 2014 | 2280 | -0.10 ± 0.03 | 248 | -0.51 ± 0.07 | -2.36 |
| Mineral soil 2 | 0-10 | September 2014 | 1150 | 0.10 ± 0.03 | 998 | -0.11 ± 0.06 | -1.96 |
| Mineral soil 2 | 10-20 | September 2014 | 1750 | 0.06 ± 0.02 | 170 | -0.71 ± 0.07 | -2.56 |
| Mineral soil 2 | 20-40 | September 2014 | 1730 | 0.07 ± 0.03 | 152 | -0.90 ± 0.06 | -2.76 |
| Mineral soil 3 | 0-10 | May 2014 | 534 | -0.42 ± 0.03 | 1210 | 0.30 ± 0.05 | -1.55 |
| Mineral soil 3 | 10-20 | May 2014 | 422 | -0.07 ± 0.03 | 83 | -1.07 ± 0.12 | -2.92 |
| Mineral soil 3 | 20-40 | May 2014 | 608 | -0.20 ± 0.02 | 55 | -1.18 ± 0.09 | -3.03 |
| Mineral soil 4 | 0-10 | May 2014 | 1270 | 0.23 ± 0.02 | 1240 | -0.48 ± 0.06 | -2.33 |
| Mineral soil 4 | 10-20 | May 2014 | 1570 | 0.09 ± 0.03 | 108 | -1.09 ± 0.07 | -2.94 |
| Mineral soil 4 | 20-40 | May 2014 | 2820 | 0.11 ± 0.03 | 107 | -1.06 ± 0.03 | -2.91 |
|  |  |  |  |  |  |  |  |
| Spruce needles 1 |  | October 2015 | 842 | -0.89 ± 0.04 | 1870 | 0.42 ± 0.09 | -1.43 |
| Spruce needles 2 |  | October 2015 | 616 | -1.26 ± 0.04 | 1510 | 0.36 ± 0.04 | -1.49 |
| Spruce needles 3 |  | October 2015 | 662 | -1.40 ± 0.04 | 1300 | 0.62 ± 0.04 | -1.23 |
| Spruce fine roots 1 |  | May 2021 | 641 | -0.21 ± 0.03 | 2860 | -0.55 ± 0.03 | -2.40 |
| Spruce fine roots 2 |  | May 2021 | 575 | -0.73 ± 0.04 | 3120 | -0.94 ± 0.02 | -2.79 |
| Spruce fine roots 3 |  | May 2021 | 597 | -0.84 ± 0.04 | 3620 | -0.58 ± 0.04 | -2.43 |
| Spruce xylem 1 |  | May 2021 | 220 | -0.33 ± 0.04 | 723 | -0.16 ± 0.03 | -2.01 |
| Spruce xylem 2 |  | May 2021 | 174 | -0.78 ± 0.04 | 764 | -0.26 ± 0.04 | -2.11 |
| Spruce xylem 3 |  | May 2021 | 369 | -0.64 ± 0.04 | 801 | 0.16 ± 0.03 | -1.69 |
| Whole rock  Sandstone I |  | May 2021 | 2410 | -0.13 ± 0.03 | 410 | 0.01 ± 0.05 | -1.84 |
| Whole rock  Sandstone II |  | May 2021 | 1800 | -0.75 ± 0.04 | 451 | 0.24 ± 0.04 | -1.61 |
| Whole rock  Sandstone III |  | May 2021 | 859 | -0.50 ± 0.04 | 177 | -0.49 ± 0.09 | -2.34 |
| Whole rock  Sandstone IV |  | May 2021 | 661 | -0.19 ± 0.03 | 94 | -0.76 ± 0.11 | -2.61 |
| Whole rock  Claystone I |  | June 2023 | 7620 | 0.28 ± 0.02 | 1460 | 0.36 ± 0.02 | -1.50 |
|  |  |  |  |  |  |  |  |
| Whole rock  Claystone II |  | June 2023 | 7680 | 0.31 ± 0.02 | 1790 | 0.19 ± 0.02 | -1.67 |
| Whole rock  Claystone III |  | June 2023 | 6580 | 0.29 ± 0.02 | 1210 | 0.24 ± 0.02 | -1.62 |
| Whole rock  Conglomerate |  | May 2021 | 640 | -0.85 ± 0.02 | 469 | 0.39 ± 0.06 | -1.47 |
