# Supplementary material for: Sizeable net export of base cations from a Carpathian flysch catchment indicates their geogenic origin while the 26Mg/24Mg, 44Ca/40Ca and 87Sr/86Sr isotope ratios in runoff are indistinguishable from atmospheric input
Source: Environ Sci Pollut Res Int. 2024 Mar 18;31(17):26261–81. doi: 10.1007/s11356-024-32866-1 (PMC11024055; doi:10.1007/s11356-024-32866-1)
Supplement: Supplementary file 13 — Supplementary file13 (DOCX 22 KB) [file 11356_2024_32866_MOESM13_ESM.docx]

**Table S2**. Ba, Rb and Sr concentrations and Sr isotope composition of selected samples.

| **Sample type** | **Depth in soil (cm)** | **Sampling date** | **Ba concentration**  **(µg L^-1^/mg kg^-1^)** | **Rb concentration**  **(µg L^-1^/mg kg^-1^)** | **Sr concentration (µg L^-1^/mg kg^-1^)** | **^87^Sr/^86^Sr** |
| --- | --- | --- | --- | --- | --- | --- |
| Open-area precipitation |  | November 2020 | 2.16 µg L^-1^ | 5.15 µg L^-1^ | 0.51 µg L^-1^ | 0.712137 ± 0.000001 |
| Spruce canopy throughfall |  | November 2020 | 2.87 | 12.5 | 0.71 | 0.714261 ± 0.000008 |
| Runoff |  | November 2020 | 36.4 | 4.85 | 25.4 | 0.715255 ± 0.000005 |
| Open-area precipitation |  | December 2020 | 1.57 | 6.36 | 0.61 | 0.71549 ± 0.000002 |
| Spruce canopy throughfall |  | December 2020 | 2.74 | 13.6 | 1.35 | 0.713168 ± 0.000005 |
| Runoff |  | December 2020 | 33.5 | 5.01 | 21.6 | 0.715228 ± 0.000002 |
| Open-area precipitation |  | January 2021 | 5.12 | 4.22 | 1.03 | 0.710470 ± 0.000001 |
| Spruce canopy throughfall |  | January 2021 | 2.67 | 3.63 | 0.65 | 0.715458 ± 0.000001 |
| Runoff |  | January 2021 | 31.4 | 6.41 | 21.4 | 0.715213 ± 0.000001 |
| Open-area precipitation |  | February 2021 | 3.21 | 3.50 | 2.09 | 0.709647 ± 0.000001 |
| Spruce canopy throughfall |  | February 2021 | 2.87 | 6.79 | 1.66 | 0.711417 ± 0.000003 |
| Runoff |  | February 2021 | 31.7 | 5.50 | 18.1 | 0.715238 ± 0.000002 |
| Open-area precipitation |  | March 2021 | 1.81 | 4.36 | 2.20 | 0.71013 ± 0.000002 |
| Spruce canopy throughfall |  | March 2021 | 5.59 | 5.91 | 3.22 | 0.711356 ± 0.000001 |
| Runoff |  | March 2021 | 32.3 | 5.92 | 17.3 | 0.715009 ± 0.000001 |
| Open-area precipitation |  | April 2021 | 1.51 | 5.38 | 0.77 | 0.711628 ± 0.000003 |
| Spruce canopy throughfall |  | April 2021 | 2.19 | 8.09 | 2.09 | 0.711741 ± 0.000001 |
| Runoff |  | April 2021 | 34.3 | 3.67 | 19.9 | 0.715268 ± 0.000001 |
| Open-area precipitation |  | May 2021 | 1.54 | 4.02 | 0.74 | 0.714151 ± 0.000001 |
| Spruce canopy throughfall |  | May 2021 | 3.34 | 12.9 | 1.79 | 0.71201 ± 0.000002 |
| Runoff |  | May 2021 | 35.5 | 5.10 | 21.9 | 0.715211 ± 0.000001 |
| Open-area precipitation |  | June 2021 | 0.80 | 4.32 | 1.85 | 0.709593 ± 0.000001 |
| Spruce canopy throughfall |  | June 2021 | 3.38 | 21.0 | 2.13 | 0.712251 ± 0.000001 |
| Runoff |  | June 2021 | 39.7 | 4.66 | 27.2 | 0.715278 ± 0.000001 |
| Open-area precipitation |  | July 2021 | 0.89 | 5.08 | 12.5 | 0.710256 ± 0.000001 |
| Spruce canopy throughfall |  | July 2021 | 4.20 | 21.0 | 2.77 | 0.712589 ± 0.000001 |
| Runoff |  | July 2021 | 42.7 | 5.72 | 30.6 | 0.715253 ± 0.000002 |
| Open-area precipitation |  | August 2021 | <0.05 | 3.02 | 0.18 | 0.712169 ± 0.000002 |
| Spruce canopy throughfall |  | August 2021 | 2.87 | 10.2 | 1.44 | 0.712787 ± 0.000001 |
| Runoff |  | August 2021 | 28.3 | 3.70 | 14.2 | 0.714845 ± 0.000002 |
| Open-area precipitation |  | September 2021 | <0.05 | 10.0 | 0.31 | 0.711933 ± 0.000002 |
| Spruce canopy throughfall |  | September 2021 | 1.92 | 17.4 | 0.86 | 0.713144 ± 0.000001 |
| Runoff |  | September 2021 | 40.07 | 12.2 | 28.3 | 0.715308 ± 0.000002 |
| Open-area precipitation |  | October 2021 | 0.45 | 9.84 | 0.58 | 0.711166 ± 0.000002 |
| Spruce canopy throughfall |  | October 2021 | 2.49 | 23.8 | 1.05 | 0.713183 ± 0.000002 |
| Runoff |  | October 2021 | 41.29 | 9.62 | 31.0 | 0.715303 ± 0.000001 |
| Soil water 1 | -60 | Jun-Aug 2021 | 93.7 | 11.3 | 10.3 | 0.714309 ± 0.000001 |
| Soil water 2 | -60 | Jun-Aug 2021 | 73.7 | 8.7 | 11.1 | 0.714646 ± 0.000001 |
| Soil water 3 | -60 | Jun-Aug 2021 | 100 | 12.4 | 20.7 | 0.712328 ± 0.000002 |
| Soil water 1 | -60 | Sep-Nov 2021 | 76.8 | 10.3 | 7.8 | 0.715453 ± 0.000011 |
|  |  |  |  |  |  |  |
| Soil water 2 | -60 | Sep-Nov 2021 | 55.7 | 8.4 | 8.9 | 0.715543 ± 0.000003 |
| Soil water 3 | -60 | Sep-Nov 2021 | 75.9 | 9.6 | 10.1 | 0.714879 ± 0.000006 |
| Mineral soil 1 | 0-10 | October 2014 | 144 mg kg^-1^ | 32.5 mg kg^-1^ | 21.1 mg kg^-1^ | 0.722645 ± 0.000002 |
| Mineral soil 1 | 10-20 | October 2014 | 228 | 72.8 | 29.9 | 0.728605 ± 0.000001 |
| Mineral soil 1 | 20-40 | October 2014 | 256 | 87.3 | 34.0 | 0.729825 ± 0.000002 |
| Mineral soil 2 | 0-10 | September 2014 | 141 | 63.3 | 25.7 | 0.722993 ± 0.000001 |
| Mineral soil 2 | 10-20 | September 2014 | 221 | 102 | 33.2 | 0.726757 ± 0.000001 |
| Mineral soil 2 | 20-40 | September 2014 | 243 | 95.3 | 33.3 | 0.727770 ± 0.000002 |
| Mineral soil 3 | 0-10 | May 2014 | 69.1 | 21.8 | 14.6 | 0.720578 ± 0.000001 |
| Mineral soil 3 | 10-20 | May 2014 | 131 | 51.1 | 19.7 | 0.727920 ± 0.000002 |
| Mineral soil 3 | 20-40 | May 2014 | 161 | 72.1 | 22.5 | 0.727732 ± 0.000002 |
| Mineral soil 4 | 0-10 | May 2014 | 207 | 73.8 | 23.8 | 0.724220 ± 0.000002 |
| Mineral soil 4 | 10-20 | May 2014 | 194 | 82.4 | 24.1 | 0.727250 ± 0.000002 |
| Mineral soil 4 | 20-40 | May 2014 | 166 | 83.2 | 25.9 | 0.727936 ± 0.000001 |
|  |  |  |  |  |  |  |
| Spruce needles 1 |  | October 2015 | 17.3 | 35.2 | 3.76 | 0.715560 ± 0.000002 |
| Spruce needles 2 |  | October 2015 | 2.87 | 32.9 | 1.38 | 0.714352 ± 0.000001 |
| Spruce needles 3 |  | October 2015 | 2.87 | 55.8 | 1.03 | 0.717075 ± 0.000001 |
| Spruce fine roots 1 |  | May 2021 | 88.6 | 13.8 | 11.1 | 0.714822 ± 0.000002 |
| Spruce fine roots 2 |  | May 2021 | 75.4 | 26.4 | 8.80 | 0.713653 ± 0.000001 |
| Spruce fine roots 3 |  | May 2021 | 47.8 | 19.4 | 6.40 | 0.71252 ± 0.000002 |
| Spruce xylem 1 |  | May 2021 | 21.6 | 2.90 | 3.66 | 0.716271 ± 0.000002 |
| Spruce xylem 2 |  | May 2021 | 9.71 | 3.51 | 3.14 | 0.726927 ± 0.000001 |
| Spruce xylem 3 |  | May 2021 | 10.1 | 4.02 | 3.13 | 0.764601 ± 0.000002 |
| Whole rock  Sandstone I |  | May 2021 | 208 | 76.8 | 26.6 | 0.726747 ± 0.000001 |
| Whole rock  Sandstone II |  | May 2021 | 209 | 86.9 | 33.3 | 0.726834 ± 0.000002 |
| Whole rock  Sandstone III |  | May 2021 | 247 | 96.8 | 30.4 | 0.732103 ± 0.000002 |
| Whole rock  Sandstone IV |  | May 2021 | 156 | 65.0 | 21.8 | 0.730545 ± 0.000002 |
| Whole rock  Claystone I |  | June 2023 | 388 | 267 | 63.5 | 0.727682 ± 0.000014 |
|  |  |  |  |  |  |  |
| Whole rock  Claystone II |  | June 2023 | 362 | 259 | 67.1 | 0.726964 ± 0.000012 |
| Whole rock  Claystone III |  | June 2023 | 481 | 242 | 56.8 | 0.729344 ± 0.000013 |
| Whole rock  Conglomerate |  | May 2021 | 65.3 | 24.5 | 13.6 | 0.718434 ± 0.000015 |
