# Supplementary material for: Sizeable net export of base cations from a Carpathian flysch catchment indicates their geogenic origin while the 26Mg/24Mg, 44Ca/40Ca and 87Sr/86Sr isotope ratios in runoff are indistinguishable from atmospheric input
Source: Environ Sci Pollut Res Int. 2024 Mar 18;31(17):26261–81. doi: 10.1007/s11356-024-32866-1 (PMC11024055; doi:10.1007/s11356-024-32866-1)
Supplement: Supplementary file 14 — Supplementary file14 (DOCX 15 KB) [file 11356_2024_32866_MOESM14_ESM.docx]

Table S3. Results of analysis of standard reference materials. SE – standard error.

|  |  | Published data^*^ | | | | | This study | | | | |
| --- | --- | --- | --- | --- | --- | --- | --- | --- | --- | --- | --- |
| Standard | Standard type | δ^25^Mg (‰) | 2 SE | δ^26^Mg (‰) | 2 SE | *n* | δ^25^Mg (‰) | 2 SE | δ^26^Mg (‰) | 2 SE | *n* |
| IAPSO | Atlantic sea water | -0.43 | 0.02 | -0.83 | 0.05 | 6 | -0.43 | 0.03 | -0.84 | 0.04 | 15 |
| NIST 1640a | Spring water | -0.38 | 0.08 | -0.73 | 0.08 | 9 | -0.38 | 0.03 | -0.79 | 0.04 | 13 |
| NIST 1515 | Apple leaves | -0.62 | 0.05 | -1.22 | 0.05 | 6 | -0.64 | 0.05 | -1.25 | 0.06 | 14 |
| NIST 2709a | Soil | -0.08 | 0.03 | -0.15 | 0.06 | 6 | -0.08 | 0.04 | -0.15 | 0.05 | 7 |
| BHVO-2 | Hawaian Basalt | -0.10 | 0.04 | -0.20 | 0.07 | 7 | -0.12 | 0.03 | -0.22 | 0.04 | 8 |
|  |  |  |  | δ^44^Ca (‰) | 2 SE | *n* |  |  | δ^44^Ca (‰) | 2 SE | *n* |
| NIST SRM 915a | Sea water |  |  | -1.86 to -1.81 |  | 3 |  |  | -1.87 | 0.07 | 4 |
| NIST SRM 915b | Sea water |  |  | -1.18 to -1.13 |  | 3 |  |  | -1.15 | 0.07 | 17 |
|  |  |  |  | ^87^Sr/^86^Sr | 2 SE | *n* |  |  | ^87^Sr/^86^Sr | 2 SE | *n* |
| NBS987 |  |  |  | 0.710241 | 12 |  |  |  | 0.710243 | 7 | 4 |
| EN-1 | Carbonate |  |  | 0.709171 | 29 | 6 |  |  | 0.709170 | 18 |  |
| BCR-2 | Basalt |  |  | 0.704920 | 1100 | 5 |  |  | 0.705015 | 8 |  |
| JG-1a | Granodiorite |  |  | 0.710979 | 37 | 10 |  |  | 0.711048 | 12 |  |
| NIST 2709a | Soil |  |  | 0.708150 |  | 2 |  |  | 0.708153 | 6 | 2 |

^*^Published data for IAPSO, NIST 1640a, NIST 1515, and NIST 2709a are after Shalev et al (2018); for BHVO-2 are after Teng et al. (2015), for NIST SRM 915a, for BCR-2, JG-1a and NISt 2709a are after GeoReM database (Jochum and Nohl, 2008), for NBS987 are after Pin et al. (2014), for EN-1 are after Erban-Kochergina et al. (2022)
